# Supplementary material for: Psychosocial impact on frontline health and social care professionals in the UK during the COVID-19 pandemic: a qualitative interview study
Source: BMJ Open. 2021 Feb 8;11(2):e047353. doi: 10.1136/bmjopen-2020-047353 (PMC7871227; doi:10.1136/bmjopen-2020-047353)
Supplement: Author's manuscript [file bmjopen-2020-047353.draft_revisions.pdf]

BMJ Open is committed to open peer review. As part of this commitment we make the peer review history of every article we publish publicly available.

When an article is published we post the peer reviewers' comments and the authors' responses online. We also post the versions of the paper that were used during peer review. These are the versions that the peer review comments apply to.

The versions of the paper that follow are the versions that were submitted during the peer review process. They are not the versions of record or the final published versions. They should not be cited or distributed as the published version of this manuscript.

BMJ Open is an open access journal and the full, final, typeset and author-corrected version of record of the manuscript is available on our site with no access controls, subscription charges or pay-per-view fees (http://bmjopen.bmj.com).

If you have any questions on BMJ Open's open peer review process please email info.bmjopen@bmj.com

BMJ Open

The psychosocial impact on frontline health and social care professionals in the UK during the COVID-19 pandemic: a qualitative interview study.

Journal:	*BMJ Open*
Manuscript ID	bmjopen-2020-047353
Article Type:	Original research
Date Submitted by the Author:	30-Nov-2020

Complete List of Authors:	Aughterson, Henry; University College London Research Department of Epidemiology and Public Health, Behavioural science and health<br>McKinlay, Alison; University College London Research Department of Epidemiology and Public Health, Department of Behavioural Science and Health<br>Fancourt, Daisy; University College London Research Department of Epidemiology and Public Health, Department of Behavioural Science and Health<br>Burton, Alexandra; University College London Research Department of Epidemiology and Public Health, Department of Behavioural Science and Health

Keywords:	MENTAL HEALTH, COVID-19, QUALITATIVE RESEARCH, Health policy < HEALTH SERVICES ADMINISTRATION & MANAGEMENT, MEDICAL EDUCATION & TRAINING, PUBLIC HEALTH

SCHOLARONE™
Manuscripts

I, the Submitting Author has the right to grant and does grant on behalf of all authors of the Work (as defined in the below author licence), an exclusive licence and/or a non-exclusive licence for contributions from authors who are: i) UK Crown employees; ii) where BMJ has agreed a CC-BY licence shall apply, and/or iii) in accordance with the terms applicable for US Federal Government officers or employees acting as part of their official duties; on a worldwide, perpetual, irrevocable, royalty-free basis to BMJ Publishing Group Ltd ("BMJ") its licensees and where the relevant Journal is co-owned by BMJ to the co-owners of the Journal, to publish the Work in this journal and any other BMJ products and to exploit all rights, as set out in our licence.

The Submitting Author accepts and understands that any supply made under these terms is made by BMJ to the Submitting Author unless you are acting as an employee on behalf of your employer or a postgraduate student of an affiliated institution which is paying any applicable article publishing charge ("APC") for Open Access articles. Where the Submitting Author wishes to make the Work available on an Open Access basis (and intends to pay the relevant APC), the terms of reuse of such Open Access shall be governed by a Creative Commons licence – details of these licences and which Creative Commons licence will apply to this Work are set out in our licence referred to above.

Other than as permitted in any relevant BMJ Author's Self Archiving Policies, I confirm this Work has not been accepted for publication elsewhere, is not being considered for publication elsewhere and does not duplicate material already published. I confirm all authors consent to publication of this Work and authorise the granting of this licence.

For peer review only

The psychosocial impact on frontline health and social care professionals in the UK during the COVID-19 pandemic: a qualitative interview study.

Henry Aughterson[1], Alison McKinlay[1], Daisy Fancourt[1], Alexandra Burton[1]

1. University College London Research Department of Epidemiology and Public Health, Behavioural science and health
London, UK

Correspondence to Henry Aughterson henry.aughterson.14@ucl.ac.uk

For peer review only

Abstract

Objectives To explore the psychosocial well-being of health and social care professionals working during the COVID-19 pandemic.

Design This was a qualitative study deploying in-depth, individual interviews, which were audio-recorded and transcribed verbatim. Thematic analysis was used for coding.

Participants This study involved 25 participants from a range of frontline professions in health and social care.

Setting Interviews were conducted over the phone or video call, depending on participant preference.

Results From the analysis, we identified 5 overarching themes: communication challenges, work-related stressors, support structures, personal growth, and individual resilience. The participants expressed difficulties such as communication challenges and changing work conditions, but also positive factors such as increased team unity at work, and a greater reflection on what matters in life.

Conclusions This study provides evidence on the support needs of health and social care professionals amid continued and future disruptions caused by the pandemic. It also elucidates some of the successful strategies (such as mindfulness, hobbies, restricting news intake, virtual socialising activities) deployed by health and social care professionals that can support their resilience and well-being and be used to guide future interventions.

Keywords COVID-19 pandemic, mental health, resilience, coping strategies, healthcare workers, carers, social workers, qualitative research, UK

Strengths and limitations of this study

- This is the first study in the UK to interview both health and social care professionals working in a range of settings on their experiences working through COVID-19.
- This study used a strong theoretical approach to inform the topic guide, and one-to-one interviews allowed in-depth analysis of the psychosocial experiences of health and social care professionals, complementing the wider availability of quantitative evidence.

For peer review only - http://bmjopen.bmj.com/site/about/guidelines.xhtml

- We interviewed a wide range of professions, which provided breadth of experience but might limit the specificity of findings.
- Given the fluctuating nature of the pandemic, attitudes of health and social care professionals may change over time. This can be challenging to capture during a single interview, however we did ask questions on how their experience had progressed longitudinally.
- Our sample may have been biased towards people who had more free time to participate and so were coping better than others. However, our sample still described a number of stressful experiences during the pandemic, and it is also possible that workers who were frustrated or stressed wished to express their views.

Introduction

To control the spread of the COVID-19 pandemic, epidemiological measures were taken across the globe, with responses differing between nations depending on their own public health circumstances, scientific advice and political priorities (1). In the UK, from March 23rd this involved a national ‘lockdown’, involving significant restrictions on citizens’ way of life including measures such as ‘staying at home’, social distancing and the closure of workplaces, shops and other services (2). Specific lockdown measures were eased over time, but major constraints and the progressive tightening and relaxing of such restraints remained for substantial periods.

Some professions, known as ‘key workers’, considered to provide an essential service to the public, were excluded from various restrictions and continued working throughout the pandemic. Crucially, health and social care professionals were designated as key workers to enable their continued support for patients and clients throughout the UK’s National Health Service (NHS) and social care system. When measures were first announced, significant concerns arose around lack of capacity within the NHS, limited personal protective equipment (PPE) and staff burnout (3). Previous research exploring the psychological impact on health and care professionals during epidemics such as SARS (Severe Acute Respiratory syndrome) and MERS (Middle East Respiratory Syndrome), has highlighted the adverse psychological effects that frontline health work during epidemics can have (4–6). There is also emerging evidence during the COVID-19 pandemic that healthcare workers experienced heightened levels of stress and anxiety (7–11), depression (8,9,12) and poor sleep quality (8,13).

There are a number of reasons why health and care workers can experience adverse psychological consequences in epidemics. First, rising cases of a new infection can lead to longer hours, more intense working environments, and work-life imbalance, which disrupt the equilibrium between work demands and workers’ response capacity (14). This, coupled with a lack of control, unclear job expectations, and lack of social support at work are the components of ‘professional burn-out’ (15). Concerns about the mental health and wellbeing of health and social care professionals in the UK were growing prior to the Covid-19 pandemic,

For peer review only - http://bmjopen.bmj.com/site/about/guidelines.xhtml

For peer review only

with ‘professional burnout’ recognised as a particular challenge (15,16). Moreover, there is evidence one may feel they lack the tools to manage (‘loss of manageability’) the confusion created by diagnosing and treating an unknown infection (‘loss of comprehensibility’) and experience a reduction of work to essential rather than meaningful patient interactions (‘loss of meaningfulness’) which combined may disrupt their ‘sense of coherence’ (SOC) (a measure of how well one is able to cope with stressors) (17,6,18,19). This disruption has been found to adversely affect mental health (the SOC theory informed part of our interview guide; See ‘Methods’) (20). However, equally there is evidence demonstrating that health and care workers have moderate to high levels of psychological resilience during times of pandemics (21), and so it is unclear whether or not they will have a robust, or disrupted, sense of coherence during COVID. Third, staff may be concerned about their own risks from exposure to a new pathogen, or the risks that they might infect family or friends. These concerns can be particularly acute when the aetiology and outcomes from a new virus are not well understood (6).

There are few published qualitative studies that have investigated the psychosocial impact of the COVID-19 pandemic on both health and social care professionals within the UK. One qualitative study interviewing 30 hospital-based healthcare workers in the UK found heightened anxiety related to PPE issues and lack of training in new skills (17), while another study with nurses and support workers in care settings identified a lack of pandemic preparedness, heightened anxiety, shortage of PPE and ever-changing PPE guidance (22). It is unclear what the psychosocial challenges faced by other health and care workers are – such as GPs, mental health and social workers. Moreover, current research on health and care workers during COVID-19 has predominantly been quantitative, using pre-assumed hypotheses of negative effects. However, in previous pandemics such as SARS and MERS, there were some positive outcomes including a more positive outlook towards work, growth under pressure, greater comradery with colleagues, and a strong sense of professional responsibility and personal development (5,6). A UK study interviewing hospital-based healthcare workers during COVID-19 also found increased solidarity between colleagues and high levels of morale (17).

There is a need for qualitative research to explore factors that may have helped alleviate distress amongst health and social care workers during the pandemic. This is crucial in order to provide richer data of their experiences to aid our understanding of specific stressors, guide future support and interventions both as COVID-19 continues, and also in the occasion of future pandemics and stressful situations within the NHS. The aims of this study were to explore: (1) The impact of the COVID-19 pandemic overall on the working lives and mental health of health and social care professionals, and (2) the factors that contribute to the resilience of health and social care professionals during the pandemic.

Methods

<u>Sample and recruitment:</u>

We recruited health and social care professionals from across the UK using social media, personal contacts, newsletters and from a sample of participants taking part in a large, nationwide, quantitative survey study: the

For peer review only - http://bmjopen.bmj.com/site/about/guidelines.xhtml

UCL COVID-19 Social Study (23). This research forms a qualitative component of this larger study. 25 participants, from a range of frontline professions within health and social care, were recruited and interviewed between May 1st and September 17th (Table 1). Sampling was purposive to include a range of ages, ethnicity, gender and professional roles. Interviews ceased when saturation was reached and the lead author identified no new themes. Presentation of recruitment, data collection and analysis are aligned with the COREQ criteria for reporting qualitative research (24).

Data collection:

Semi-structured, one-to-one, telephone or video interviews were conducted by HA (PhD student and trainee medic) and AB (mental health services researcher) exploring the impact of the pandemic on participants' social lives, work life, and mental health. The interviews lasted an average of 51 minutes (range 29-93). Berkman's social networks framework and Antonovsky's sense of coherence (SOC) theory informed the topic guide questions on social life and mental health, respectively (25,20). The full Topic Guide is provided in Supplementary Material and exemplary questions are provided in Figure 1. All participants were given a Participant Information Sheet and encouraged to ask questions. Written informed consent was then obtained and a demographics form completed by all participants. We audio-recorded interviews with participants' consent, and recordings were transcribed by a professional transcription service.

[Figure 1 to go ~ here]

Figure 1: Examples of Questions in the Topic Guide

Patient and public involvement

The study participants or public were not involved in the design of the study, the conduct of the study, the writing of the paper nor in the dissemination of the study results. However, participants will be sent study results if requested, and the findings will be shared with the wider public through newsletters (the MARCH network) and social media.

Data analysis:

The analytical approach deployed was reflexive thematic analysis (26). This followed the steps described by Braun and Clarke (27) of familiarisation with the data, generation and definition of codes, theme searching, and producing the report. HA and AM (research psychologist) independently coded four transcripts, which were discussed before HA coded and interpreted all remaining transcripts, continuing until saturation was reached and no new codes identified. A deductive approach was used to develop the initial coding framework based on concepts in the topic guide, followed by an inductive coding approach as new concepts were added to the framework based on the data. Contradictory data and context around codes was retained, to capture subtle nuances. Codes were then grouped into themes, with each theme representing a meaningful pattern in

For peer review only - http://bmjopen.bmj.com/site/about/guidelines.xhtml

the data. All final themes were agreed by study authors. Weekly team meetings with researchers from the qualitative COVID-19 Social Study team were also utilised to discuss and develop findings. The software utilised for coding was NVivo qualitative data analysis, Version 12 (28).

Results

We interviewed 25 participants, from a range of professions within health and social care including doctors, nurses, carers and social workers working in hospital, residential, community and primary care settings. Participants were aged 26-65, predominantly female (80%) and White British (68%).

Table 1: Characteristics of the health and social care professionals

Number of participants	25
Profession	Hospital doctor (6)<br>GP (4)<br>Hospital nurse (3)<br>Social worker (3)<br>Home carer (2)<br>Care home carer (2)<br>Assistant psychologist (1)<br>Community nurse (1)<br>Practice nurse (1)<br>Counsellor & psychotherapist (1)<br>Academic physiotherapist (1)
Age	Range 26-65
Gender	Male 5<br>Female 20
Ethnicity	White British 17<br>Asian 3<br>Black British 2<br>White & Asian 1<br>White Irish 1<br>White Other 1

<u>Themes:</u>

5 primary themes were identified. These were: **Communication challenges**, **Work-related stressors**, **Support structures**, **Resilience**, and **Personal growth**. Themes and corresponding sub-themes are displayed in Figure 1.

For peer review only - http://bmjopen.bmj.com/site/about/guidelines.xhtml

[Figure 2 to go ~ here]

Figure 2: Themes and sub-themes

Communication challenges

The pandemic brought with it numerous challenges around communication for health and social care professionals in their work, particularly when moving consultations online and having difficult discussions.

Virtual consulting

Some consultations had shifted online, especially among GPs, therapists and social workers. Several participants said that one of the key benefits was the increased efficiency of virtual communication and consultations.

"much more is done remotely, which is just so much more efficient, because patients don't always need to be seen face to face. Patients preferred it, we preferred it." (Participant_16_GP)

However, more often participants talked about the limitations of virtual consultations. Some found it difficult to provide appropriate emotional support, especially in a time where there was heightened need. This was more commonly an issue for social care, mental health and palliative care professionals supporting vulnerable adults or children.

"It makes me realise the importance of seeing someone face to face to actually support them. I just don't think a telephone call or a Zoom call is sufficient when it comes to helping people who have profound mental health issues, or even mild mental health issues. And I think that some people just need the power of touch or a hug or a face-to-face human person to ensure that they're kept safe and okay" (Participant_4_Hospital doctor_palliative_care_registrar)

Participants often found it difficult to build new relationships virtually, especially when working with children:

"I think if you already know someone well, and you are speaking to them on video call, then that's fine because you've already established the relationship… but if you have to start establishing the relationship on a video call…there is something missing I think, especially working with children… especially if you have a child that's introverted or struggles to communicate or has learning difficulties or is very shy. It's harder to make them feel comfortable when you're on video call" (Participant_10_social_worker)

For peer review only - http://bmjopen.bmj.com/site/about/guidelines.xhtml

Participants talked about difficulties identifying crucial signs of deterioration in health from patients or clients, e.g. from body language:

“we have to be able to pick up signs, for instance, if they are suicidal, I think there’s an anxiety there that doing it online, it’s difficult sometimes to do that, to pick up on some nuances of the way they talk… we can’t see their whole body language” (Participant_9_counsellor&psychotherapist)

Participants, especially mental health and social workers, experienced difficulty ensuring a confidential space when consulting virtually:

“When they’re (children) talking to you, perhaps you see them in a room and it seems like they’re alone. But actually, maybe they have all their family members that are standing in the corner” (Participant_10_social_worker)

Difficult conversations

A common challenge during this period was an increase in difficult conversations with patients, clients and their family members. The need for PPE, and virtual consultations, accentuated these difficulties. This was especially apparent in those working in services that provide mental health and palliative care.

“Family communication is awful these days… You can’t see someone, and you’re speaking to them on the phone, and you’re telling them that they can’t come and see their loved one, and that their loved one might well die, that’s an awful conversation to have with someone…it’s definitely one of the worst things about COVID for me” (Participant_8_Hospital_doctor)

Several participants said they were providing more emotional support to patients and clients than usual:

“There’s been a huge amount of emotional support that we’ve had to give through anxiety, through grief. All that has been heightened quite greatly really. And a deeper sense of sadness in yourself, that you’re trying to support people and having that empathy for them, thinking this is just absolutely horrendous for them” (Participant_20_Home_carer)

Work-related stressors

Health and social care professionals experienced a range of challenging emotions and psychological difficulties.

For peer review only - http://bmjopen.bmj.com/site/about/guidelines.xhtml

The need to protect loved ones

Nearly all participants spoke about ongoing worry for friends and family compounded by a fear of transmitting the virus to them – due to participants' increased risk of catching it at work.

"Normally I would see my mum every day… but where I work, I was worried, I was more at risk to catch anything, so I definitely didn't. My sisters were actually going into the garden, and talking to her… but I wouldn't, so I was standing at the gate. So that was pretty hard, because I'm very close to my mum" (Participant_21_Care_home_manager&carer)

Increased workload and changing work conditions

Fatigue and exhaustion were commonly reported by participants throughout this period.

"I'm just feeling really run down… I literally have 4 or 5 weeks where I've not left the house unless it's just to pop to the supermarket… I find that really does impact me… it's like work has taken over my whole life and I'm exhausted" (Participant_11_Family_support_worker)

Whilst not unanimous, some participants experienced longer working hours and increased workload.

"my routine was really like… wake up, eat something, go into work, which as shifts as nurses we had to stay in the hospital for 12 and a half hours…go home and eat something, drink something, go to sleep… then wake up and then go to work again… we have been extremely busy compared to the normality" (Participant_19_Hospital_nurse)

Feelings of fatigue were also enhanced by the tenuous nature of PPE:

"you just become quite tired…it culminated with masks, visors, aprons, hot weather and regulations changing and sometimes you'd come home from a shift and feel you'd been pulled in all directions really" (Participant_20_Home_carer&nurse)

Public not following the rules

Some participants experienced frustration with members of the public not following social distancing and other guidelines, feeling that their work on the frontline was being undermined.

"I've been quite annoyed… you're trying your best in lockdown to obey the government guidelines and I think I've had a huge amount of frustration by hearing and seeing people who haven't…certain politicians as well that haven't stuck to guidelines, and I feel sometimes I've been working my socks off and felt quite cheated" (Participant_20_Home_carer&nurse)

For peer review only - http://bmjopen.bmj.com/site/about/guidelines.xhtml

Uncertainty of risk to patients

A common concern was making decisions that balanced the complex, and often unknown, risks associated with the virus along with other health risks. This was particularly difficult during the early phases of the pandemic, when less was known about the virus.

"…sometimes I've thought, right, we do need to bring patients in, but then are you putting them at more risk exposing them to the virus, which could actually kill them, and actually they could potentially just have a gastro bug and not bowel cancer?" (Participant_16_GP)

Support structures

The availability of support structures at work and home was identified as an important buffer for the psychosocial impact of working during the pandemic and in coping with considerable work-related changes.

Team unity:

Many participants felt closer to their team, and that team unity had increased during the pandemic, united over a common cause. This was more likely if teams were cohesive before the pandemic, and was particularly apparent for doctors in primary and secondary care.

"just having the vibes that we're all in this together and we're all going (through) the same thing, and we're pulling in the same direction" (Participant_3_Hospital_doctor_intensive_care)

Some of this unity was facilitated through virtual communication:

"We actually started up a group, ourselves, on WhatsApp. It's just our team, it doesn't include management. It's just for family support workers and social workers… we try not to put work stuff on there… we try to send each other funny messages or memes… to keep us going" (Participant_11_family_support_worker)

Some participants felt the increase in virtual meetings improved attendance, due to the ease of just being able to "dial in" and improved collaborative working among multidisciplinary teams.

"when we're safeguarding a child, you are supposed to work collaboratively… it'll be social care and school, health, maybe a youth organisation or domestic abuse organisation… when you're doing these physical meetings, beforehand, people just wouldn't turn up… but now, they can just dial in" (Participant_23_Social_worker)

For peer review only - http://bmjopen.bmj.com/site/about/guidelines.xhtml

However, team unity was not unanimous, with several participants experiencing loneliness, due to an increase in lone working or working from home.

"there was a time…I was then at home isolating for two weeks, and so I was working from home. I had remote access to my computer, so I was doing purely telephone consultations from home and I felt very isolated there, and I didn't feel like I was part of the team at all" (Participant_13_GP)

A few participants also described difficulties connecting with colleagues virtually for support:

"I think it could have been improved by seeing each other face-to-face, and I have to say that has been really detrimental to our team. I think you lose a lot by not seeing someone face to face, in terms of their body language and non-verbal cues aren't always captured particularly well through IT" (Participant_4_Hospital_doctor_palliative_care_registrar)

Leadership

Participants expressed frustration about government handling and changing advice throughout the pandemic. In particular, participants talked about confusing guidance received from management and government regarding PPE or distancing procedures at work, as well as the speed in which the guidance changed.

"I know they (upper management in NHS) have difficult decisions to make quickly but I sometimes find their rule making quite vague. A bit like the government, I feel like they're making it up as they go along somewhat. And it changed every day so you'd log onto your emails and there'd be some new change" (Participant_15_community_mental_health_nurse)

However, some felt the culture of blame itself was frustrating:

"the other thing that annoys me sometimes is that... everyone wants to blame everybody…it's like the blame game…everybody has to blame Boris… Somebody's responsible. China's responsible... I feel that illnesses and viruses have been around forever and ever and ever… you can't really be pointing the finger all the time, and I find it quite depressing" (Participant_12_Hospital_doctor_critical_care)

Most participants felt supported at work and received regular emotional and practical check-ins from management. A small number felt ignored by management, which led to feelings of being overwhelmed:

"I was quite anxious about being in the office with COVID... I had some colleagues of mine who were able to work from home… I was told that this wasn't possible… it was business as usual. It was a real sense of

For peer review only - http://bmjopen.bmj.com/site/about/guidelines.xhtml

frustration, not feeling that you're being listened to by my manager and just a sense of feeling overwhelmed and quite helpless about the situation" (Participant_24_Assistant_psychologist)

Social support

Most participants had supportive relationships with family, friends and colleagues, which helped contribute to a sense of resilience among participants.

"I've got a good, strong marriage and we're a good partnership, my husband and I, and we've supported each other through all this… our friends are going through similar things, so I'm able to talk to my friends as well. I've got a good network of friends, so I'm very lucky." (Participant_20_Home_carer&nurse)

They also felt supported by their local community, and the public, e.g. through the 'Clap for Carers' movement where people across the country stood outside their front door once a week to applaud health and care workers for their contribution:

"the Clap for Carers thing, the neighbours would come out and clap, and I found that quite touching… quite uplifting actually" (Participant_12_Hospital_doctor_critical_care)

Resilience

Despite the difficulties and challenges, most participants said they experienced psychosocial benefits during the pandemic, which many attributed to their individual coping style or sense of resilience.

Proactive coping

Most participants said they used well-developed coping mechanisms to deal with the ever-changing circumstances, including engaging in hobbies, participating in virtual activities, and maintaining routines.

"I do genuinely believe getting out into nature has a really good impact on one's mental health and it certainly does on mine…Crafting, doing things, keeping busy…that's really important for your mental health having that occupations. I crochet… we paint, we draw, we make jewellery…"
(Participant_15_Community_mental_health_nurse)

Many participants discussed the negative impact of constant news coverage on coronavirus and the death count, and intentionally restricted their news intake as they felt it was unhelpful, even harmful to their mental health:

For peer review only

For peer review only - http://bmjopen.bmj.com/site/about/guidelines.xhtml

"the virus messes with your head more than it does your body, if you're not hospitalised. That's just down to the media at the end of the day. There's so much media and so much emphasis on death, not so much on recovery" (Participant_7_Hospital_nurse)

Accepting uncertainty

Most participants had a degree of personal, psychological resilience linked to an acceptance, or 'letting go', of what they had no control over, such as the overall outcome of the pandemic or government restrictions.

"I'm generally much less anxious now than I was in January and February. And part of that, I think, is about thinking a lot more about death and being a lot more accepting about death, and about what you can control and what's out of your control" (Participant_14_GP)

Part of this philosophy seemed to be influenced by their previous experiences, and their profession:

"Because of my job, I think I'm aware that we're not really in control of lots of things in our life… I see that all the time with patients and people I care for… we don't have control over everything and we have to have a level of acceptance for that" (Participant_15_community_mental_health_nurse)

Increased sense of purpose and reward

Most participants expressed gratitude for being able to continue working. This brought with it benefits such as purpose, daily structure, predictability, a degree of socialising and being 'in-the-know' about the virus. Participants also talked about how it felt good to be able to contribute. For many, this was highly rewarding and brought a heightened sense of purpose.

"…some people would view me as not being lucky, because I'm a frontline worker, but that's my job and that's what I'm trained to do, so I don't view that as unlucky… I have been lucky in the sense that I've been able to keep busy, to keep working, to feel I'm contributing" (Participant_17_GP)

Guilt occurred when participants weren't able to contribute as much as they would have liked e.g. they were told they couldn't work because they were high-risk, or had to work from home.

"at first, I felt incredibly guilty by the fact that I wasn't helping out on the frontline because I was pregnant. I was just told I'm not allowed to see any patients, by occupational health, and sent home" (Participant_6_Hospital_doctor)

Personal growth

The pandemic also brought with it opportunities for personal growth among participants.

For peer review only - http://bmjopen.bmj.com/site/about/guidelines.xhtml

Increased reflection

Many participants experienced a greater reflection on “what matters” in their lives. Commonly this was spending quality time with friends and family and appreciating the small things in life.

“one benefit is that actually we quite like a simple life and actually you come to appreciate the very simple things, which are just being outside, going for a bike ride, having a picnic…just the health and happiness of your own family is what’s important and everything else, you can generally sort out” (Participant_17_GP)

Slowing down

Many participants discussed how the pandemic had given them a chance to slow down and have more “me-time” and expressed this was something they wished to take forward beyond the pandemic. This view may seem in conflict with the increased workload experienced by some participants, but reflected the changes outside of work, e.g. having fewer social obligations due to social distancing restrictions.

“We have really busy lives generally, and we spend a lot of time rushing around doing lots of stuff, and actually this time has been quite nice in many ways as a period to kind of slow down a bit and I think just appreciating each other” (Participant_1_Hospital_doctor)

Improved non-work relationships

Whilst not unanimous, many participants talked about how some of their relationships with others improved during the pandemic, especially with family members they lived with, the crisis having brought them closer.

“I’ve connected with my family a lot more… I feel really good about spending more time with my daughter…that’s time that I would never have had with her, so that’s really special” (Participant_5_academic_respiratory_physiotherapist)

Discussion

This qualitative interview study explored the psychosocial impact of the COVID-19 pandemic on health and social care professionals in the UK. We identified five key themes shared between professionals’ accounts. The main difficulties reported were ‘communication challenges’ (consisting of ‘virtual consulting’ and ‘difficult conversations’) and ‘work-related stressors’ (consisting of ‘need to protect loved ones’, ‘public not following rules’, ‘increased workload and changing work conditions’ and ‘uncertainty of risk to patients’). Three factors appeared to mitigate some of the psychological distress of the pandemic: ‘support structures’ (consisting of ‘team unity’, ‘leadership’ and ‘social support’), ‘resilience’ (consisting of ‘proactive coping’, ‘accepting uncertainty’ and ‘increased sense of purpose and reward’) and ‘personal growth’ (consisting of ‘slowing down’, ‘increased reflection’ and ‘improved relationships’).

For peer review only - http://bmjopen.bmj.com/site/about/guidelines.xhtml

The themes were drawn from interviews with professionals working in different areas of health and social care, and some themes were felt more strongly in certain jobs than others. For example, GPs and social workers enjoyed the efficiency of new virtual consultations, but GPs found it difficult to use with older adults and those with age-related cognitive decline. Those working with mental health clients, such as psychotherapists and social workers, experienced digital connectivity issues when communicating with vulnerable clients, leading to frustrating repetitions and difficulties building a trusting relationship. This corroborates findings from previous research on barriers to virtual consulting (29). Those working with older adults, found one of the most challenging elements of the pandemic was having difficult conversations over the telephone with loved ones of patients who were dying - often having to convey that visiting restrictions meant they would be unable to say goodbye in person. This is potentially concerning for the well-being of patients and healthcare professionals, given previous research has highlighted the importance of an appropriate physical and social setting in breaking bad news, and also the presence of family members (30,31).

There were a number of common work-related stressors among participants. One of these was frustration with members of the public not following the social distancing and hygiene regulations, and with the government's handling of the pandemic. Some also expressed frustration at the 'culture of blame' that they felt permeated the media and public discourse, which can be maladaptive and harmful for one's own mental well-being (32). Emotional and physical fatigue were also common experiences across all professions, corroborating qualitative studies of health and care workers during COVID-19 and previous pandemics globally (4,5,33). Many participants were worried about putting their loved ones at risk of catching COVID-19 as highlighted by previous research (34), however in our study it appears to have been a particular concern amongst carers and hospital doctors/nurses, perhaps due to their higher level of virus exposure.

Most participants experienced an increased sense of team unity; that they were 'all in this together' fighting a common enemy. This may have led to a degree of resilience against some of the stressful elements of working in a pandemic, supporting findings from previous pandemics (5,10). Moreover, supporting Berkman's social networks theory (35) which informed part of our interview guide, strong social relationships were frequently cited by participants as key supportive mechanisms for their mental health during this period, including supportive partners at home, friends, family and colleagues. Those participants that did experience loneliness at work during the pandemic were lone workers, working from home, or described unsupportive management. In line with our findings, a recent systematic review of quantitative studies examining the impact of COVID-19 on healthcare workers found social support to be a vital resource underlying their ability to cope (21).

Participants also described using their own internal resilience as a way to buffer many of the key stressors involved in working through the pandemic, and even thrive at times. They adopted proactive coping mechanisms, as seen in healthcare workers during SARS (36). These often involved partaking in activities that

For peer review only - http://bmjopen.bmj.com/site/about/guidelines.xhtml

now have substantial evidence for their role in improving mental health including: exercise (37), arts and crafts (38), spending time in nature (39,40), virtual activities with friends, and maintaining a healthy routine (41,42). Consistent with research from previous pandemics and recent quantitative data during COVID-19, participants also restricted their news intake, particularly as they felt the constant reporting of COVID-19 and the prevalent discourse of blame negatively impacted their mental health (6,23).

Most participants said they were successfully able to ‘let go’ of aspects of the pandemic that they felt were out of their control, such as the overall course of the virus and government restrictions. This demonstrates a psychological theory known as ‘radical acceptance’ (43), and may have been responsible in part for the resilience reported by many participants, also having been identified as a successful coping strategy for healthcare workers in previous pandemics (36). Some academics have critiqued ‘resilience’ as a concept for its focus on individual- rather than structural-level factors (44), however, participants in the study highlighted the link between these factors, particularly the importance of social networks and social support structures at work. Most participants also expressed gratitude for being able to continue working and described a sense of increased purpose and reward for being able to contribute during the pandemic.

Common ‘personal growth’ themes were frequently described in participant accounts. Most participants reflected more on ‘what matters’ in life during this period, which included relationships with friends and family, their health and the health of loved ones, and ‘appreciating the small things’ in life. These findings mirror international qualitative studies looking at the psychological impact of COVID-19 and previous pandemics on health and care workers which found they experienced ‘growth under pressure’ (5,33) and increased gratitude and self-reflection (33). ‘Growth under pressure’ may be a closely linked (but slightly diluted) concept to ‘post-traumatic growth’ seen in individuals experiencing personal growth in the aftermath of highly challenging life crises (45). Lastly, participants demonstrated a high “sense of coherence” which enhanced their ability to cope during stressful experiences (19). Participants spoke about ‘manageability’ whereby they were highly proactive in their coping mechanisms, ‘comprehensibility’ in their enhanced understanding of the virus and need for social distancing restrictions, and ‘meaningfulness’ in how they experienced a heightened sense of purpose through their contribution during the pandemic.

Strengths & Limitations

This is the first study in the UK to interview both health and social care professionals working in a range of settings on their experiences working through COVID-19, which we felt important as they all continued to provide vital frontline care during the pandemic. This study used a strong theoretical approach to inform the topic guide, and one-to-one interviews allowed in-depth analysis of the psychosocial experiences of health and social care professionals, complementing the wider breadth of quantitative evidence. There were also some limitations. First, we interviewed a wide range of professions, which provided breadth of experience but might limit the specificity of findings. However, due to similarities in the roles of health and care professionals we felt

For peer review only - http://bmjopen.bmj.com/site/about/guidelines.xhtml

it important to include a range of voices. Second, given the fluctuating nature of the pandemic, attitudes of health and social care professionals may change over time. This can be difficult to capture during a single interview, however we did ask questions on how their experience had progressed longitudinally. Third, our sample may have been biased towards people who had more free time to participate and so were coping better than others. However, our sample still described a number of stressful experiences during the pandemic, and it is equally possible that workers who were frustrated or stressed wished to express their views.

Implications

This study has important implications for health and social care workers, managers, commissioners of services and policy makers during the ongoing pandemic and beyond. First, it highlights the key stressors experienced by health and social care professionals during the COVID-19 pandemic. Many of these echo findings from previous epidemics, but whilst this is reassuring in terms of data credibility, it highlights a concerning lack of improvement in working conditions during such emergencies over the past two decades. It is vital that the challenges identified here are addressed. Health and care professionals navigating difficult conversations via telephone or video may benefit from extra training and support at work, for example in use of the WIRE-SPIKES protocol for breaking bad news remotely (46). Further, this study provides evidence for the supportive and coping mechanisms used by workers who experienced resilience during this period. Application of coping strategies including leisure activities were common and reportedly beneficial, as were the use of mindful techniques such as expressing gratitude. This suggests that health and care professionals may benefit from regular work-based interventions providing space for such activities. Whilst such activities may feel extraneous during emergency situations, the building of resilience and positive coping outside of pandemic situations and the tackling of problems such as staff burnout will likely improve staff coping capacity in future epidemic situations. Alongside this, adequate provision for social support should be ensured, from family and friends but also via the work place e.g. through enhanced supervision or peer support. The research presented here suggests that investment into wellbeing support could play a vital role in helping health and care workers to manage emotional stress.

Conclusion

To the best of our knowledge, this is the first qualitative study to explore the psychosocial impact of the COVID-19 pandemic on both health and social care professionals working in different settings across the UK. Participants experienced communication challenges and changing work conditions, but also positive factors such as increased team unity, and greater reflection on what matters in their life. This study offers important evidence for continued and future disruptions caused by the COVID-19 pandemic. It also elucidates successful psychological and practical strategies deployed by health and social care professionals that could be used to support their resilience and well-being.

For peer review only - http://bmjopen.bmj.com/site/about/guidelines.xhtml

For peer review only

Funding statement

This COVID-19 Social Study was funded by the Nuffield Foundation [WEL/FR-000022583], but the views expressed here are those of the authors. The study was also supported by the MARCH Mental Health Network funded by the Cross-Disciplinary Mental Health Network Plus initiative supported by UK Research and Innovation [ES/S002588/1], and by the Wellcome Trust [221400/Z/20/Z]. DF was funded by the Wellcome Trust [205407/Z/16/Z].

Ethical approval

The study was reviewed and approved by the UCL Ethics Committee (Project ID 14895/005).

Acknowledgements

The authors would like to thank other members of the qualitative COVID-19 social study team at UCL, who helped discuss themes during the analysis stages at weekly team meetings: Dr Thomas May, Dr Anna Roberts, and Dr Joanna Dawes.

Contributors: DF, AB and HA conceived the study and contributed to the study design. HA conducted all the interviews apart from 1, which AB conducted. HA coded all the transcriptions. AM coded 4 transcripts for cross-checking purposes. HA wrote up the manuscript. All authors (HA, DF, AM, AB) critically reviewed the manuscript and approved the final submission.

Competing interests: None declared.

Data sharing statement: Raw data is available via participant quotations and the topic interview guide is provided in Supplementary Material.

References

1. Adhikari SP, Meng S, Wu Y-J, Mao Y-P, Ye R-X, Wang Q-Z, et al. Epidemiology, causes, clinical manifestation and diagnosis, prevention and control of coronavirus disease (COVID-19) during the early outbreak period: a scoping review. Infect Dis Poverty. 2020 Mar 17;9(1):29.

For peer review only - http://bmjopen.bmj.com/site/about/guidelines.xhtml

2. Iacobucci G. Covid-19: UK lockdown is "crucial" to saving lives, say doctors and scientists. BMJ [Internet]. 2020 Mar 24 [cited 2020 Sep 3];368. Available from: https://www.bmj.com/content/368/bmj.m1204

3. Shaw SCK. Hopelessness, helplessness and resilience: The importance of safeguarding our trainees' mental wellbeing during the COVID-19 pandemic. Nurse Educ Pract. 2020 Mar;44:102780.

4. McAlonan GM, Lee AM, Cheung V, Cheung C, Tsang KWT, Sham PC, et al. Immediate and sustained psychological impact of an emerging infectious disease outbreak on health care workers. Can J Psychiatry. 2007 Apr;52(4):241–7.

5. Kim Y. Nurses' experiences of care for patients with Middle East respiratory syndrome-coronavirus in South Korea. American Journal of Infection Control. 2018 Jul 1;46(7):781–7.

6. Lee S-H, Juang Y-Y, Su Y-J, Lee H-L, Lin Yi-Hui, Chao C-C. Facing SARS: psychological impacts on SARS team nurses and psychiatric services in a Taiwan general hospital. General Hospital Psychiatry. 2005 Sep 1;27(5):352–8.

7. Kang L, Li Y, Hu S, Chen M, Yang C, Yang BX, et al. The mental health of medical workers in Wuhan, China dealing with the 2019 novel coronavirus. The Lancet Psychiatry. 2020 Mar 1;7(3):e14.

8. Lai J, Ma S, Wang Y, Cai Z, Hu J, Wei N, et al. Factors Associated With Mental Health Outcomes Among Health Care Workers Exposed to Coronavirus Disease 2019. JAMA Netw Open. 2020 Mar 23;3(3):e203976.

9. Du J, Dong L, Wang T, Yuan C, Fu R, Zhang L, et al. Psychological symptoms among frontline healthcare workers during COVID-19 outbreak in Wuhan. Gen Hosp Psychiatry [Internet]. 2020 Apr 3 [cited 2020 Oct 26]; Available from: https://www.ncbi.nlm.nih.gov/pmc/articles/PMC7194721/

10. Sun N, Wei L, Shi S, Jiao D, Song R, Ma L, et al. A qualitative study on the psychological experience of caregivers of COVID-19 patients. American Journal of Infection Control. 2020 Jun 1;48(6):592–8.

11. Xiao H, Zhang Y, Kong D, Li S, Yang N. The Effects of Social Support on Sleep Quality of Medical Staff Treating Patients with Coronavirus Disease 2019 (COVID-19) in January and February 2020 in China. Med Sci Monit. 2020 Mar 5;26:e923549-1-e923549-8.

12. Lu W, Wang H, Lin Y, Li L. Psychological status of medical workforce during the COVID-19 pandemic: A cross-sectional study. Psychiatry Res. 2020 Jun;288:112936.

13. Huang Y, Zhao N. Generalized anxiety disorder, depressive symptoms and sleep quality during COVID-19 outbreak in China: a web-based cross-sectional survey. Psychiatry Res. 2020 Jun;288:112954.

14. Quick TL. Linking productivity and health. Healthy Work: Stress, Productivity, and the Reconstruction of Working Life Robert Karasek and Tores Theorell New York: Basic Books, Inc. 1990 $29.95 Canada $39.95 381 pages. National Productivity Review. 1990;9(4):475–8.

15. Imo UO. Burnout and psychiatric morbidity among doctors in the UK: A systematic literature review of prevalence and associated factors. BJPsych Bulletin. 2017 Aug;41(4):197–204.

16. Coyle D, Edwards D, Hannigan B, Fothergill A, Burnard P. A systematic review of stress among mental health social workers. International Social Work. 2005 Mar 1;48(2):201–11.

17. Vindrola-Padros C, Andrews L, Dowrick A, Djellouli N, Fillmore H, Gonzalez EB, et al. Perceptions and experiences of healthcare workers during the COVID-19 pandemic in the UK. BMJ Open. 2020 Nov 1;10(11):e040503.

18. Williamson V, Murphy D, Greenberg N. COVID-19 and experiences of moral injury in front-line key workers. Occup Med (Lond). 2020 Jul 17;70(5):317–9.

For peer review only - http://bmjopen.bmj.com/site/about/guidelines.xhtml

19. Eriksson M. The Sense of Coherence in the Salutogenic Model of Health. In: Mittelmark MB, Sagy S, Eriksson M, Bauer GF, Pelikan JM, Lindström B, et al., editors. The Handbook of Salutogenesis [Internet]. Cham (CH): Springer; 2017 [cited 2019 Nov 27]. Available from: http://www.ncbi.nlm.nih.gov/books/NBK435812/

20. Eriksson M, Lindstrom B. Antonovsky's sense of coherence scale and its relation with quality of life: a systematic review. Journal of Epidemiology & Community Health. 2007 Nov 1;61(11):938–44.

21. Labrague LJ. Psychological resilience, coping behaviours, and social support among healthcare workers during the COVID-19 pandemic: a systematic review of quantitative studies. medRxiv. 2020 Nov 6;2020.11.05.20226415.

22. Nyashanu M, Pfende F, Ekpenyong M. Exploring the challenges faced by frontline workers in health and social care amid the COVID-19 pandemic: experiences of frontline workers in the English Midlands region, UK. Journal of Interprofessional Care. 2020 Sep 2;34(5):655–61.

23. Bu F, Steptoe A, Mak HW, Fancourt D. Time-use and mental health during the COVID-19 pandemic: a panel analysis of 55,204 adults followed across 11 weeks of lockdown in the UK. medRxiv. 2020 Aug 21;2020.08.18.20177345.

24. Tong A, Sainsbury P, Craig J. Consolidated criteria for reporting qualitative research (COREQ): a 32-item checklist for interviews and focus groups. Int J Qual Health Care. 2007 Dec 1;19(6):349–57.

25. Berkman LF. The Assessment of Social Networks and Social Support in the Elderly. Journal of the American Geriatrics Society. 1983;31(12):743–9.

26. Braun V, Clarke V. Reflecting on reflexive thematic analysis. Qualitative Research in Sport, Exercise and Health. 2019 Aug 8;11(4):589–97.

27. Braun V, Clarke V. Using thematic analysis in psychology. Qualitative Research in Psychology. 2006 Jan;3(2):77–101.

28. QSR International Pty Ltd. NVIVO [Internet]. [cited 2020 Nov 4]. Available from: https://www.qsrinternational.com/nvivo-qualitative-data-analysis-software/home

29. Donaghy E, Atherton H, Hammersley V, McNeilly H, Bikker A, Robbins L, et al. Acceptability, benefits, and challenges of video consulting: a qualitative study in primary care. Br J Gen Pract. 2019 Sep 1;69(686):e586–94.

30. Monden KR, Gentry L, Cox TR. Delivering Bad News to Patients. Baylor University Medical Center Proceedings. 2016 Jan 1;29(1):101–2.

31. Ptacek JT, Fries EA, Eberhardt TL, Ptacek JJ. Breaking bad news to patients: physicians' perceptions of the process. Support Care Cancer. 1999 Apr 1;7(3):113–20.

32. Tennen H, Affleck G. Blaming others for threatening events. Psychological Bulletin. 1990;108(2):209–32.

33. Sun N, Wei L, Shi S, Jiao D, Song R, Ma L, et al. A qualitative study on the psychological experience of caregivers of COVID-19 patients. American Journal of Infection Control. 2020 Jun 1;48(6):592–8.

34. Balasubramanian A, Paleri V, Bennett R, Paleri V. Impact of COVID-19 on the mental health of surgeons and coping strategies. Head & Neck. 2020;42(7):1638–44.

35. Berkman LF. Social Networks and Health. 2010;27.

For peer review only - http://bmjopen.bmj.com/site/about/guidelines.xhtml

36. Wong TW, Yau JKY, Chan CLW, Kwong RSY, Ho SMY, Lau CC, et al. The psychological impact of severe acute respiratory syndrome outbreak on healthcare workers in emergency departments and how they cope. European Journal of Emergency Medicine. 2005 Feb;12(1):13–18.

37. Stathopoulou G, Powers MB, Berry AC, Smits JAJ, Otto MW. Exercise Interventions for Mental Health: A Quantitative and Qualitative Review. Clinical Psychology: Science and Practice. 2006;13(2):179–93.

38. Fancourt D, Finn S. What is the evidence on the role of the arts in improving health and well-being? A scoping review [Internet]. WHO Regional Office for Europe, Copenhagen; 2019. (WHO Health Evidence Network Synthesis Reports). Available from: http://europepmc.org/books/NBK553773

39. Clatworthy J, Hinds J, Camic PM. Gardening as a mental health intervention: a review. Mental Health Review Journal [Internet]. 2013 Nov 29 [cited 2020 Oct 30]; Available from: https://www.emerald.com/insight/content/doi/10.1108/MHRJ-02-2013-0007/full/html

40. Kotera Y, Richardson M, Sheffield D. Effects of Shinrin-Yoku (Forest Bathing) and Nature Therapy on Mental Health: a Systematic Review and Meta-analysis. Int J Ment Health Addiction [Internet]. 2020 Jul 28 [cited 2020 Oct 30]; Available from: https://doi.org/10.1007/s11469-020-00363-4

41. Lyall LM, Wyse CA, Graham N, Ferguson A, Lyall DM, Cullen B, et al. Association of disrupted circadian rhythmicity with mood disorders, subjective wellbeing, and cognitive function: a cross-sectional study of 91 105 participants from the UK Biobank. The Lancet Psychiatry. 2018 Jun 1;5(6):507–14.

42. Lanza HI, Drabick DAG. Family Routine Moderates the Relation Between Child Impulsivity and Oppositional Defiant Disorder Symptoms. J Abnorm Child Psychol. 2011 Jan 1;39(1):83–94.

43. Robins CJ, Schmidt III H, Linehan MM. Dialectical Behavior Therapy: Synthesizing Radical Acceptance with Skillful Means. In: Mindfulness and acceptance: Expanding the cognitive-behavioral tradition. New York, NY, US: Guilford Press; 2004. p. 30–44.

44. Fletcher D, Sarkar M. Psychological resilience: A review and critique of definitions, concepts, and theory. European Psychologist. 2013;18(1):12–23.

45. Tedeschi RG, Calhoun LG. Posttraumatic Growth: Conceptual Foundations and Empirical Evidence. Psychological Inquiry. 2004 Jan 1;15(1):1–18.

46. Holstead RG, Robinson AG. Discussing Serious News Remotely: Navigating Difficult Conversations During a Pandemic. JCO Oncology Practice. 2020 May 18;16(7):363–8.

47. Kinsella P, Garland A. Cognitive Behavioural Therapy for Mental Health Workers: A Beginner's Guide. Routledge; 2008. 265 p.

48. Shapiro SL, Brown KW, Biegel GM. Teaching self-care to caregivers: Effects of mindfulness-based stress reduction on the mental health of therapists in training. Training and Education in Professional Psychology. 2007;1(2):105–15.

49. Cocchiara RA, Peruzzo M, Mannocci A, Ottolenghi L, Villari P, Polimeni A, et al. The Use of Yoga to Manage Stress and Burnout in Healthcare Workers: A Systematic Review. Journal of Clinical Medicine. 2019 Mar;8(3):284.

For Peer Review Only

For peer review only - http://bmjopen.bmj.com/site/about/guidelines.xhtml

- How would you describe your social life now that social distancing measures have been brought in because of Covid-19?
- In what ways has your work life been impacted by the Covid-19 pandemic?
- How do you feel about the changes that have been brought about by Covid-19? Have they had any impact on your mental health or wellbeing?
- Have there been any positive experiences for you resulting from the Covid-19 pandemic?

Figure 1: Examples of Questions in the Topic Guide

For peer review only

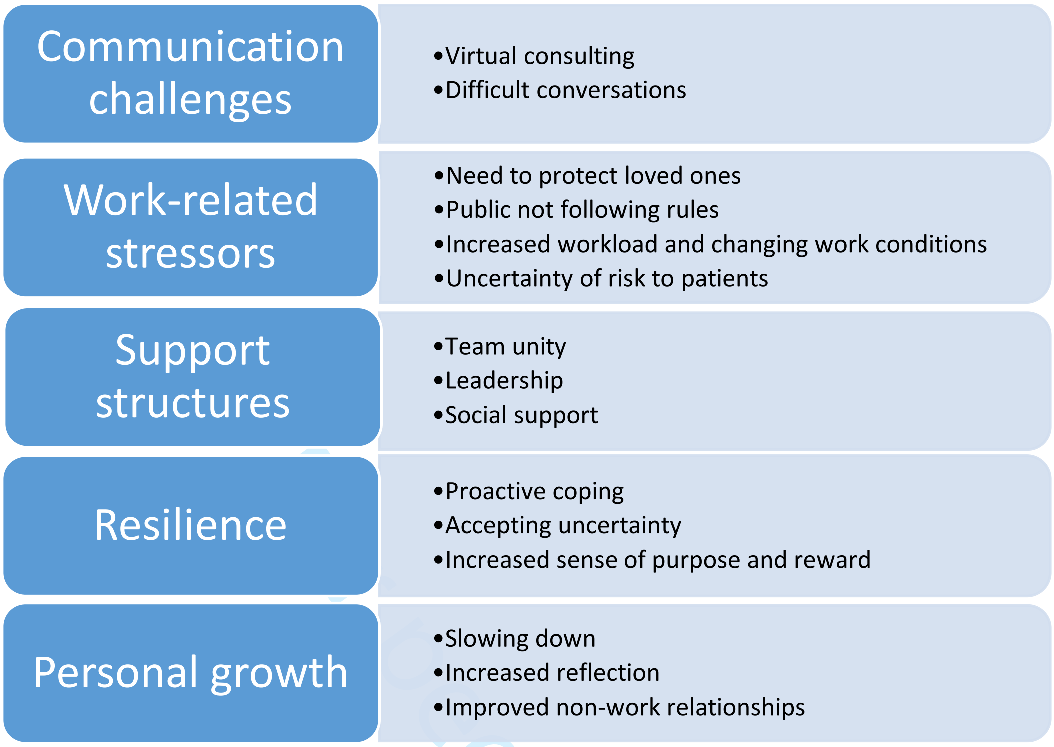

Figure 2: Themes and sub-themes

Draft topic guide: Health and Care Workers

Ask to describe 'normal life' – before the crisis, and now

- Employed? Type of job, hours etc,
- Full time parent or carer?
- Who you normally live with, does this change, separated/ extended family?
- Whether you would usually have done any type(s) of regular exercise (whatever they perceive as exercise including walking/gardening)

SOCIAL LIFE

What was your social life before the Covid-19 pandemic? Has this changed? If so, what has been the impact of Covid-19 on your social life?

- How would you describe your social network before Covid – for example size, types of people, types of relationships, do they live with you, nearby or further away, how often do you see each other, how well do you know each other? How do you interact, face to face, online or social media? Describe some of your common socialising activities. **Has this changed? What has the impact of Covid been on your social network?**
- Can you tell us about any ways your social networks/ friendship groups influence you, such as peer pressure, or encouraging you to get involved in things? Do you compare your life to theirs?
- Could you describe any community participation or volunteering participation before Covid? **Has this changed? If so, what has been the impact of Covid-19 on community participation/volunteering participation?**
- Could you describe the social support you have before Covid? (such as emotional support, advice and information, someone to help you with money or milk/bread/essentials) **Has this changed? If so, what has been the impact of Covid-19 on your social support?**
- **Social engagement (social roles, bonding, attachment) (pre- and post- Covid)**

For peer review only

WORK LIFE

How would you describe your work life before the Covid-19 pandemic?
Prompts include:

- Describe a typical day?
- Describe your work environment prior to the crisis
- How much autonomy did you have in your role?
- Did you find your job rewarding?
- Did you feel able to do your job to a high standard?
- Did you enjoy your job?
- Describe your sense, if any, of team unity or disunity prior to this crisis?
- How able were you to follow organisational rules and how did you feel about this?
- Normally did you feel safe at work? In what way?

How would you describe your work life since the Covid-19 pandemic? Please tell us about this

For peer review only - http://bmjopen.bmj.com/site/about/guidelines.xhtml

- Describe a typical day now – how have common work practices changed? Have you adapted your work in response to Covid-19 (e.g. delivery, operating hours, change of products/production methods)
- Describe your overall work environment now
- How much autonomy do you feel you have at the moment and how has this changed?
- Are you finding work rewarding at the moment?
- Do you feel able to do your job to a high standard – has this changed since the crisis?
- Enjoyment – do you currently enjoy your job?
- Describe your sense, if any, of team unity or disunity during this crisis?
- How able are you to follow organisational rules and how do you feel about this?
- Do you feel safe? If this has changed, how?

MENTAL HEALTH

How do you feel about the changes that have been brought about by Covid-19? Have they had any impact on your mental health or wellbeing? Please tell us about these

- What are the things most bothering you at the moment (work or outside of work)?
- What have been the major triggers/causes of any mental health or wellbeing issues?
- How have government guidelines or organisational guidelines impacted your mental health or wellbeing?
- Have you experienced any impact on positive emotions? (prompts: how deeply you can engage with what you are doing, sense of meaning/ purpose, relationships with others, how well you are managing and feelings of control over your situation?)
- Has there been any impact on your sense of identity?
- Have you experienced any negative psychological feelings? (prompts: such as shame, guilt, lack of pleasure, anxiety, worry)
- Please tell us about any physical symptoms due to being stressed or anxious? (prompts: fatigue, sleep problems, pain, illness symptoms, palpitations)

Have you been doing/ planning anything to help with this?

- How has your support been, from friends/family? From work colleagues/your organisation?
- Connecting with family or friends online
- Online groups?
- Hobbies/ Reading
- Exercise at home <ask about what they have been doing and if there are specific resources they have found useful to exercise>
- Volunteering
- Other engagement

Why are you doing/ not doing these things?

- Helpful/ not helpful – please tell us why
- Enjoyable
- Good for mental health/ wellbeing
- Can't get online, not connected, not comfortable, affordability, confidence in using/ skills
- Skills in using the internet/ communication software
- Living arrangements/ Work/ caring demands
- Peer support/ pressure
- Difficulties/ restriction in physical environment

PROSPECTION

For peer review only - http://bmjopen.bmj.com/site/about/guidelines.xhtml

Has the pandemic meant that you have any worries for the future?

- Worries about work/the future of your work?
- Worries for yourself? Anything not directly connected to work?

How are these different from the worries you had before?

- Sense of control/ powerlessness
- Severity of worries / perspective

Will this change the way you live your life in future?

- The way you connect with others
- How you look after yourself
- How you support others
- How you exercise?

Do you think there will be any changes to the way you work in the future? Why/why not?

Has this changed any of your priorities for the future?

For peer review only

For peer review only - http://bmjopen.bmj.com/site/about/guidelines.xhtml

COREQ (COnsolidated criteria for REporting Qualitative research) Checklist

A checklist of items that should be included in reports of qualitative research. You must report the page number in your manuscript where you consider each of the items listed in this checklist. If you have not included this information, either revise your manuscript accordingly before submitting or note N/A.

Topic	Item No.	Guide Questions/Description	Reported on Page No.
**Domain 1: Research team and reflexivity**			
Personal characteristics			
Interviewer/facilitator	1	Which author/s conducted the interview or focus group?	
Credentials	2	What were the researcher's credentials? E.g. PhD, MD	
Occupation	3	What was their occupation at the time of the study?	
Gender	4	Was the researcher male or female?	
Experience and training	5	What experience or training did the researcher have?	
Relationship with participants			
Relationship established	6	Was a relationship established prior to study commencement?	
Participant knowledge of the interviewer	7	What did the participants know about the researcher? e.g. personal goals, reasons for doing the research	
Interviewer characteristics	8	What characteristics were reported about the inter viewer/facilitator? e.g. Bias, assumptions, reasons and interests in the research topic	
**Domain 2: Study design**			
Theoretical framework			
Methodological orientation and Theory	9	What methodological orientation was stated to underpin the study? e.g. grounded theory, discourse analysis, ethnography, phenomenology, content analysis	
Participant selection			
Sampling	10	How were participants selected? e.g. purposive, convenience, consecutive, snowball	
Method of approach	11	How were participants approached? e.g. face-to-face, telephone, mail, email	
Sample size	12	How many participants were in the study?	
Non-participation	13	How many people refused to participate or dropped out? Reasons?	
Setting			
Setting of data collection	14	Where was the data collected? e.g. home, clinic, workplace	
Presence of non-participants	15	Was anyone else present besides the participants and researchers?	
Description of sample	16	What are the important characteristics of the sample? e.g. demographic data, date	
Data collection			
Interview guide	17	Were questions, prompts, guides provided by the authors? Was it pilot tested?	
Repeat interviews	18	Were repeat inter views carried out? If yes, how many?	
Audio/visual recording	19	Did the research use audio or visual recording to collect the data?	
Field notes	20	Were field notes made during and/or after the inter view or focus group?	
Duration	21	What was the duration of the inter views or focus group?	
Data saturation	22	Was data saturation discussed?	
Transcripts returned	23	Were transcripts returned to participants for comment and/or	

For peer review only

Topic	Item No.	Guide Questions/Description	Reported on Page No.
		correction?	
**Domain 3: analysis and findings**			
Data analysis			
Number of data coders	24	How many data coders coded the data?	
Description of the coding tree	25	Did authors provide a description of the coding tree?	
Derivation of themes	26	Were themes identified in advance or derived from the data?	
Software	27	What software, if applicable, was used to manage the data?	
Participant checking	28	Did participants provide feedback on the findings?	
Reporting			
Quotations presented	29	Were participant quotations presented to illustrate the themes/findings? Was each quotation identified? e.g. participant number	
Data and findings consistent	30	Was there consistency between the data presented and the findings?	
Clarity of major themes	31	Were major themes clearly presented in the findings?	
Clarity of minor themes	32	Is there a description of diverse cases or discussion of minor themes?	

Developed from: Tong A, Sainsbury P, Craig J. Consolidated criteria for reporting qualitative research (COREQ): a 32-item checklist for interviews and focus groups. *International Journal for Quality in Health Care*. 2007. Volume 19, Number 6: pp. 349 – 357

Once you have completed this checklist, please save a copy and upload it as part of your submission. DO NOT include this checklist as part of the main manuscript document. It must be uploaded as a separate file.

For peer review only

BMJ Open

The psychosocial impact on frontline health and social care professionals in the UK during the COVID-19 pandemic: a qualitative interview study.

Journal:	*BMJ Open*
Manuscript ID	bmjopen-2020-047353.R1
Article Type:	Original research
Date Submitted by the Author:	14-Jan-2021
Complete List of Authors:	Aughterson, Henry; University College London Research Department of Epidemiology and Public Health, Behavioural science and health<br>McKinlay, Alison; University College London Research Department of Epidemiology and Public Health, Department of Behavioural Science and Health<br>Fancourt, Daisy; University College London Research Department of Epidemiology and Public Health, Department of Behavioural Science and Health<br>Burton, Alexandra; University College London Research Department of Epidemiology and Public Health, Department of Behavioural Science and Health
**Primary Subject Heading**:	Qualitative research
Secondary Subject Heading:	Epidemiology, Health policy, Mental health, Public health, Sociology
Keywords:	MENTAL HEALTH, COVID-19, QUALITATIVE RESEARCH, Health policy < HEALTH SERVICES ADMINISTRATION & MANAGEMENT, MEDICAL EDUCATION & TRAINING, PUBLIC HEALTH

SCHOLARONE™
Manuscripts

I, the Submitting Author has the right to grant and does grant on behalf of all authors of the Work (as defined in the below author licence), an exclusive licence and/or a non-exclusive licence for contributions from authors who are: i) UK Crown employees; ii) where BMJ has agreed a CC-BY licence shall apply, and/or iii) in accordance with the terms applicable for US Federal Government officers or employees acting as part of their official duties; on a worldwide, perpetual, irrevocable, royalty-free basis to BMJ Publishing Group Ltd ("BMJ") its licensees and where the relevant Journal is co-owned by BMJ to the co-owners of the Journal, to publish the Work in this journal and any other BMJ products and to exploit all rights, as set out in our licence.

The Submitting Author accepts and understands that any supply made under these terms is made by BMJ to the Submitting Author unless you are acting as an employee on behalf of your employer or a postgraduate student of an affiliated institution which is paying any applicable article publishing charge ("APC") for Open Access articles. Where the Submitting Author wishes to make the Work available on an Open Access basis (and intends to pay the relevant APC), the terms of reuse of such Open Access shall be governed by a Creative Commons licence – details of these licences and which Creative Commons licence will apply to this Work are set out in our licence referred to above.

Other than as permitted in any relevant BMJ Author's Self Archiving Policies, I confirm this Work has not been accepted for publication elsewhere, is not being considered for publication elsewhere and does not duplicate material already published. I confirm all authors consent to publication of this Work and authorise the granting of this licence.

For peer review only

The psychosocial impact on frontline health and social care professionals in the UK during the COVID-19 pandemic: a qualitative interview study.

Henry Aughterson[1], Alison McKinlay[1], Daisy Fancourt[1], Alexandra Burton[1]

1. University College London Research Department of Epidemiology and Public Health, Behavioural science and health
London, UK

Correspondence to Henry Aughterson henry.aughterson.14@ucl.ac.uk 07534241106 1-19 Torrington Place WC1E 7HB

For peer review only

Abstract

Objectives To explore the psychosocial well-being of health and social care professionals working during the COVID-19 pandemic.

Design This was a qualitative study deploying in-depth, individual interviews, which were audio-recorded and transcribed verbatim. Thematic analysis was used for coding.

Participants This study involved 25 participants from a range of frontline professions in health and social care.

Setting Interviews were conducted over the phone or video call, depending on participant preference.

Results From the analysis, we identified 5 overarching themes: communication challenges, work-related stressors, support structures, personal growth, and individual resilience. The participants expressed difficulties such as communication challenges and changing work conditions, but also positive factors such as increased team unity at work, and a greater reflection on what matters in life.

Conclusions This study provides evidence on the support needs of health and social care professionals amid continued and future disruptions caused by the pandemic. It also elucidates some of the successful strategies (such as mindfulness, hobbies, restricting news intake, virtual socialising activities) deployed by health and social care professionals that can support their resilience and well-being and be used to guide future interventions.

Keywords COVID-19 pandemic, mental health, resilience, coping strategies, healthcare workers, carers, social workers, qualitative research, UK

Strengths and limitations of this study

- This is the first known study in the UK to interview both health and social care professionals working in a range of settings on their experiences working through COVID-19.

For peer review only - http://bmjopen.bmj.com/site/about/guidelines.xhtml

- This study used a strong theoretical approach to inform the topic guide, and one-to-one interviews allowed in-depth analysis of the psychosocial experiences of health and social care professionals, complementing the wider availability of quantitative evidence.
- We interviewed a wide range of professions, which provided breadth of experience but might limit the specificity of findings.
- Given the fluctuating nature of the pandemic, attitudes of health and social care professionals may change over time. This can be challenging to capture during a single interview, however we did ask questions on how their experience had progressed longitudinally.
- Our sample may have been biased towards people who had more free time to participate and so were coping better than others. However, our sample still described a number of stressful experiences during the pandemic, and it is also possible that workers who were frustrated or stressed wished to express their views.

Introduction

To control the spread of the COVID-19 pandemic, epidemiological measures were taken across the globe, with responses differing between nations depending on their own public health circumstances, scientific advice and political priorities (1). In the UK, from March 23rd this involved a national 'lockdown', involving significant restrictions on citizens' way of life including measures such as 'staying at home', social distancing and the closure of workplaces, shops and other services (2). Specific lockdown measures were eased over time, but major constraints and the progressive tightening and relaxing of such restraints remained for substantial periods.

Some professions, known as 'key workers', considered to provide an essential service to the public, were excluded from various restrictions and continued working throughout the pandemic. Crucially, health and social care professionals were designated as key workers to enable their continued support for patients and clients throughout the UK's National Health Service (NHS) and social care system. When measures were first announced, significant concerns arose around lack of capacity within the NHS, limited personal protective equipment (PPE) and staff burnout (3). Previous research exploring the psychological impact on health and care professionals during epidemics such as SARS (Severe Acute Respiratory syndrome) and MERS (Middle East Respiratory Syndrome), has highlighted the adverse psychological effects that frontline health work during epidemics can have (4–6). There is also emerging evidence during the COVID-19 pandemic that healthcare workers experienced heightened levels of stress and anxiety (7–11), depression (8,9,12) and poor sleep quality (8,13).

There are a number of reasons why health and care workers can experience adverse psychological consequences in epidemics. First, rising cases of a new infection can lead to longer hours, more intense working environments, and work-life imbalance, which disrupt the equilibrium between work demands and

For peer review only - http://bmjopen.bmj.com/site/about/guidelines.xhtml

workers' response capacity (14). This, coupled with a lack of control, unclear job expectations, and lack of social support at work are the components of 'professional burn-out' (15). Concerns about the mental health and wellbeing of health and social care professionals in the UK were growing prior to the Covid-19 pandemic, with 'professional burnout' recognised as a particular challenge (15,16). Moreover, there is evidence one may feel they lack the tools to manage ('loss of manageability') the confusion created by diagnosing and treating an unknown infection ('loss of comprehensibility') and experience a reduction of work to essential rather than meaningful patient interactions ('loss of meaningfulness') which combined may disrupt their 'sense of coherence' (SOC) (a measure of resilience – how effectively one is able to cope with stressors) (17,6,18,19). This disruption has been found to adversely affect mental health (the SOC theory informed part of our interview guide; See 'Methods') (20). However, equally there is evidence demonstrating that health and care workers have moderate to high levels of psychological resilience during times of pandemics (21), and so it is unclear whether or not they will have a robust, or disrupted, sense of coherence during COVID. Third, staff may be concerned about their own risks from exposure to a new pathogen, or the risks that they might infect family or friends. These concerns can be particularly acute when the aetiology and outcomes from a new virus are not well understood (6).

There are few published qualitative studies that have investigated the psychosocial impact of the COVID-19 pandemic on both health and social care professionals within the UK. One qualitative study interviewing 30 hospital-based healthcare workers in the UK found heightened anxiety related to PPE issues and lack of training in new skills (17), while another study with nurses and support workers in care settings identified a lack of pandemic preparedness, heightened anxiety, shortage of PPE and ever-changing PPE guidance (22). It is unclear what the psychosocial challenges faced by other health and care workers are – such as GPs, mental health and social workers. Moreover, current research on health and care workers during COVID-19 has predominantly been quantitative, using pre-assumed hypotheses of negative effects. However, in previous pandemics such as SARS and MERS, there were some positive outcomes including a more positive outlook towards work, growth under pressure, greater comradery with colleagues, and a strong sense of professional responsibility and personal development (5,6). A UK study interviewing hospital-based healthcare workers during COVID-19 also found increased solidarity between colleagues and high levels of morale (17).

There is a need for qualitative research to explore factors that have helped to alleviate distress and build resilience amongst health and social care workers during the pandemic. This is crucial in order to provide richer data of their experiences to aid our understanding of specific stressors, guide future support and interventions both as COVID-19 continues, and also in the occasion of future pandemics and stressful situations within the NHS. There may be common factors that contribute towards individuals' resilience during this period which may provide useful learning for employers and employees in order to better harness or encourage such factors. Therefore, the aims of this study were to explore: (1) The impact of the COVID-19 pandemic overall on the working lives and mental health of health and social care professionals, and (2) the

For peer review only - http://bmjopen.bmj.com/site/about/guidelines.xhtml

factors that help alleviate distress and contribute to the resilience of health and social care professionals during the pandemic.

Methods

Sample and recruitment:

We recruited health and social care professionals from across the UK using social media, personal contacts, newsletters and from a sample of participants taking part in a large, nationwide, quantitative survey study: the UCL COVID-19 Social Study (23). This research forms a qualitative component of this larger study. 25 participants, from a range of frontline professions within health and social care, were recruited and interviewed between May 1st and September 17th (Table 1). Sampling was purposive to include a range of ages, ethnicity, gender and professional roles. Interviews ceased when data sufficiency was reached and the lead author identified no new themes. Presentation of recruitment, data collection and analysis are aligned with the COREQ criteria for reporting qualitative research (24).

Data collection:

Semi-structured, one-to-one, telephone or video interviews were conducted by HA (PhD student and trainee medic) and AB (mental health services researcher) exploring the impact of the pandemic on participants' social lives, work life, and mental health. The interviews lasted an average of 51 minutes (range 29-93). Berkman's social networks framework and Antonovsky's sense of coherence (SOC) theory informed the topic guide questions on social life and mental health, respectively – e.g. including questions on social networks, social roles and meaning/purpose that related to each theory (25,20). The full Topic Guide is provided in Supplementary Material and exemplary questions are provided in Figure 1. All participants were given a Participant Information Sheet and encouraged to ask questions. Written informed consent was then obtained and a demographics form completed by all participants. We audio-recorded interviews with participants' consent, and recordings were transcribed by a professional transcription service. The audio recording of interviews and available transcripts enabled the repeating revisiting of data in order to remain true to participants' original accounts, helping to enhance validity of the results.

[Figure 1 to go ~ here]

Figure 1: Examples of Questions in the Topic Guide

Patient and public involvement

The study participants or public were not involved in the design of the study, the conduct of the study, the writing of the paper nor in the dissemination of the study results. However, participants will be sent study results if requested, and the findings will be shared with the wider public through newsletters (the MARCH network) and social media.

For peer review only - http://bmjopen.bmj.com/site/about/guidelines.xhtml

Data analysis:

The analytical approach deployed was reflexive thematic analysis (26). This followed the steps described by Braun and Clarke (27) of familiarisation with the data, generation and definition of codes, theme searching, and producing the report. HA and AM (research psychologist) independently coded four transcripts, which were discussed before HA coded and interpreted all remaining transcripts, continuing until data sufficiency was reached and no new codes identified. A deductive approach was used to develop the initial coding framework based on concepts in the topic guide, followed by an inductive coding approach (where new themes were generated from our data) as new concepts were added to the framework based on the data. Contradictory data and context around codes was retained, to capture subtle nuances. Codes were then grouped into themes, with each theme representing a meaningful pattern in the data. All final themes were agreed by study authors. We have included rich and verbatim descriptions of participants' accounts in order to support these findings. Weekly team meetings with researchers from the qualitative COVID-19 Social Study team were also utilised to discuss and develop findings. This method helped to reduce individual-level research bias that may have affected the interpretation of results, thus enhancing validity of the findings. The software utilised for coding was NVivo qualitative data analysis, Version 12 (28).

Results

We interviewed 25 participants, from a range of professions within health and social care including doctors, nurses, carers and social workers working in hospital, residential, community and primary care settings. Participants were aged 26-65, predominantly female (80%) and White British (68%).

Table 1: Characteristics of the health and social care professionals

Number of participants	25
Profession	Hospital doctor (6)<br>GP (4)<br>Hospital nurse (3)<br>Social worker (3)<br>Home carer (2)<br>Care home carer (2)<br>Assistant psychologist (1)<br>Community nurse (1)<br>Practice nurse (1)<br>Counsellor & psychotherapist (1)<br>Academic physiotherapist (1)
Age	Range 26-65

For peer review only - http://bmjopen.bmj.com/site/about/guidelines.xhtml

Gender	Male 5<br>Female 20
Ethnicity	White British 17<br>Asian 3<br>Black British 2<br>White & Asian 1<br>White Irish 1<br>White Other 1

Themes:

5 primary themes were identified. These were: **Communication challenges**, **Work-related stressors**, **Support structures**, **Resilience**, and **Personal growth**. Themes and corresponding sub-themes are displayed in Figure 2.

[Figure 2 to go ~ here]

Figure 2: Themes and sub-themes

Communication challenges

The pandemic brought with it numerous challenges around communication for health and social care professionals in their work. One of these was associated with new virtual means of consulting, and the other with the greater levels of difficult conversations.

Virtual consulting

Some consultations had shifted online, especially among GPs, therapists and social workers. Several participants said that one of the key benefits was the increased efficiency of virtual communication and consultations.

"Much more is done remotely, which is just so much more efficient, because patients don't always need to be seen face to face. Patients preferred it, we preferred it." (Participant_16_GP)

However, more often participants talked about the limitations of virtual consultations. Participants found it difficult to provide appropriate emotional support, especially in a time where there was heightened need. This was more commonly an issue for social care, mental health and palliative care professionals supporting vulnerable adults or children.

For peer review only - http://bmjopen.bmj.com/site/about/guidelines.xhtml

"It makes me realise the importance of seeing someone face to face to actually support them. I just don't think a telephone call or a Zoom call is sufficient when it comes to helping people who have profound mental health issues, or even mild mental health issues. And I think that some people just need the power of touch or a hug or a face-to-face human person to ensure that they're kept safe and okay" (Participant_4_Hospital doctor_palliative_care_registrar)

Participants often found it difficult to build new relationships virtually, especially when working with children:

"I think if you already know someone well, and you are speaking to them on video call, then that's fine because you've already established the relationship… but if you have to start establishing the relationship on a video call…there is something missing I think, especially working with children… especially if you have a child that's introverted or struggles to communicate or has learning difficulties or is very shy. It's harder to make them feel comfortable when you're on video call" (Participant_10_social_worker)

Participants talked about difficulties identifying crucial signs of deterioration in health from patients or clients, e.g. from body language:

"We have to be able to pick up signs, for instance, if they are suicidal, I think there's an anxiety there that doing it online, it's difficult sometimes to do that, to pick up on some nuances of the way they talk… we can't see their whole body language" (Participant_9_counsellor&psychotherapist)

Participants, especially mental health and social workers, reported difficulty ensuring a confidential space when consulting virtually:

"When they're (children) talking to you, perhaps you see them in a room and it seems like they're alone. But actually, maybe they have all their family members that are standing in the corner" (Participant_10_social_worker)

Difficult conversations

A common challenge during this period was an increase in difficult conversations with patients, clients and their family members. The need for PPE, and virtual consultations, accentuated these difficulties. This was especially apparent in those working in services that provide mental health and palliative care.

"Family communication is awful these days… You can't see someone, and you're speaking to them on the phone, and you're telling them that they can't come and see their loved one, and that their loved one might well die, that's an awful conversation to have with someone…it's definitely one of the worst things about COVID for me" (Participant_8_Hospital_doctor)

For peer review only - http://bmjopen.bmj.com/site/about/guidelines.xhtml

Several participants said they were providing more emotional support to patients and clients than usual:

“There’s been a huge amount of emotional support that we’ve had to give through anxiety, through grief. All that has been heightened quite greatly really. And a deeper sense of sadness in yourself, that you’re trying to support people and having that empathy for them, thinking this is just absolutely horrendous for them” (Participant_20_Home_carer)

Work-related stressors

Health and social care professionals said that they experienced a range of challenging emotions and psychological difficulties. This included frustration at members of public not following social distancing rules, the concern about protecting their loved ones from infection, and increased workload and changing working conditions.

Public not following the rules

Participants said that they felt frustrated with members of the public not following social distancing and other guidelines, feeling that their work on the frontline was being undermined.

“I’ve been quite annoyed… you’re trying your best in lockdown to obey the government guidelines and I think I’ve had a huge amount of frustration by hearing and seeing people who haven’t…certain politicians as well that haven’t stuck to guidelines, and I feel sometimes I’ve been working my socks off and felt quite cheated” (Participant_20_Home_carer&nurse)

“I didn’t see my boyfriend for six months. I just did not want to leave my house because of how I feared and felt that other people were behaving… just seeing people not following the rules” (Participant_24_Assistant_psychologist)

The need to protect loved ones

Nearly all participants spoke about ongoing worry for friends and family compounded by a fear of transmitting the virus to them – due to participants’ increased risk of catching it at work. Participants reported taking extra measures in order to protect their loved ones.

“Normally I would see my mum every day… but where I work, I was worried, I was more at risk to catch anything, so I definitely didn’t. My sisters were actually going into the garden, and talking to her… but I wouldn’t, so I was standing at the gate. So that was pretty hard, because I’m very close to my mum” (Participant_21_Care_home_manager&carer)

For peer review only - http://bmjopen.bmj.com/site/about/guidelines.xhtml

For peer review only

"It would be super lovely to have a giant hug from my dad, but I know that's not possible... in the line of work that I do, the risk to him would just be immense because I have been on the COVID wards" (Participant_4_Hospital_doctor_palliative_care_registrar)

Increased workload and changing work conditions

Fatigue and exhaustion were commonly reported by participants throughout this period.

"I'm just feeling really run down... I literally have 4 or 5 weeks where I've not left the house unless it's just to pop to the supermarket... I find that really does impact me... it's like work has taken over my whole life and I'm exhausted" (Participant_11_Family_support_worker)

Whilst not unanimous, some participants said that they were working longer hours and had an increased workload.

"My routine was really like... wake up, eat something, go into work, which as shifts as nurses we had to stay in the hospital for 12 and a half hours...go home and eat something, drink something, go to sleep... then wake up and then go to work again... we have been extremely busy compared to the normality" (Participant_19_Hospital_nurse)

Feelings of fatigue were also enhanced by the tenuous nature of PPE:

"you just become quite tired...it culminated with masks, visors, aprons, hot weather and regulations changing and sometimes you'd come home from a shift and feel you'd been pulled in all directions really" (Participant_20_Home_carer&nurse)

Moreover, a common concern for participants was making decisions that balanced the complex, and often unknown, risks associated with the virus along with other health risks. This was particularly difficult during the early phases of the pandemic, when less was known about the virus.

"...sometimes I've thought, right, we do need to bring patients in, but then are you putting them at more risk exposing them to the virus, which could actually kill them, and actually they could potentially just have a gastro bug and not bowel cancer?" (Participant_16_GP)

Support structures

The availability of support structures at work and home was identified as an important buffer for the psychosocial impact of working during the pandemic and in coping with considerable work-related changes.

For peer review only - http://bmjopen.bmj.com/site/about/guidelines.xhtml

Themes associated with this feeling of being supported included team unity and leadership at work, and wider social support.

Team unity:

Participants often felt they were closer to their team, and that team unity had increased during the pandemic, united over a common cause. This was more likely if teams were cohesive before the pandemic, and was particularly apparent for doctors in primary and secondary care.

"just having the vibes that we're all in this together and we're all going (through) the same thing, and we're pulling in the same direction" (Participant_3_Hospital_doctor_intensive_care)

Some of this unity was facilitated through virtual communication:

"We actually started up a group, ourselves, on WhatsApp. It's just our team, it doesn't include management. It's just for family support workers and social workers… we try not to put work stuff on there… we try to send each other funny messages or memes… to keep us going" (Participant_11_family_support_worker)

Participants felt the increase in virtual meetings improved attendance, due to the ease of just being able to "dial in" and improved collaborative working among multidisciplinary teams.

"When we're safeguarding a child, you are supposed to work collaboratively… it'll be social care and school, health, maybe a youth organisation or domestic abuse organisation… when you're doing these physical meetings, beforehand, people just wouldn't turn up… but now, they can just dial in" (Participant_23_Social_worker)

However, team unity was not unanimous, with several participants experiencing loneliness, due to an increase in lone working or working from home.

"I was then at home isolating for two weeks, and so I was working from home. I had remote access to my computer, so I was doing purely telephone consultations from home and I felt very isolated there, and I didn't feel like I was part of the team at all" (Participant_13_GP)

A few participants also described difficulties connecting with colleagues virtually for support:

"I think it could have been improved by seeing each other face-to-face, and I have to say that has been really detrimental to our team. I think you lose a lot by not seeing someone face to face, in terms of their body

For peer review only - http://bmjopen.bmj.com/site/about/guidelines.xhtml

language and non-verbal cues aren't always captured particularly well through IT" (Participant_4_Hospital_doctor_palliative_care_registrar)

Leadership

Participants expressed frustration about government handling and changing advice throughout the pandemic. In particular, participants talked about confusing guidance received from management and government regarding PPE or distancing procedures at work, as well as the speed in which the guidance changed.

"I know they (upper management in NHS) have difficult decisions to make quickly but I sometimes find their rule making quite vague. A bit like the government, I feel like they're making it up as they go along somewhat. And it changed every day so you'd log onto your emails and there'd be some new change" (Participant_15_community_mental_health_nurse)

However, some felt the culture of blame itself was frustrating:

"the other thing that annoys me sometimes is that... everyone wants to blame everybody…it's like the blame game…everybody has to blame Boris… Somebody's responsible. China's responsible... I feel that illnesses and viruses have been around forever and ever and ever… you can't really be pointing the finger all the time, and I find it quite depressing" (Participant_12_Hospital_doctor_critical_care)

Most participants felt supported at work and received regular emotional and practical check-ins from management. A small number felt ignored by management, which led to feelings of being overwhelmed:

"I was quite anxious about being in the office with COVID... I had some colleagues of mine who were able to work from home… I was told that this wasn't possible… it was business as usual. It was a real sense of frustration, not feeling that you're being listened to by my manager and just a sense of feeling overwhelmed and quite helpless about the situation" (Participant_24_Assistant_psychologist)

Social support

Most participants had supportive relationships with family, friends and colleagues, which helped contribute to a sense of resilience among participants.

"I've got a good, strong marriage and we're a good partnership, my husband and I, and we've supported each other through all this… our friends are going through similar things, so I'm able to talk to my friends as well. I've got a good network of friends, so I'm very lucky." (Participant_20_Home_carer&nurse)

For peer review only

They also felt supported by their local community, and the public, e.g. through the 'Clap for Carers' movement where people across the country stood outside their front door once a week to applaud health and care workers for their contribution:

"the Clap for Carers thing, the neighbours would come out and clap, and I found that quite touching… quite uplifting actually" (Participant_12_Hospital_doctor_critical_care)

Resilience

Despite the difficulties and challenges, participants also demonstrated significant psychological resilience. This was often to do with their proactive coping mechanisms, their ability to 'accept uncertainty' and the increased sense of purpose associated with their work.

Proactive coping

Most participants said they used well-developed coping mechanisms to deal with the ever-changing circumstances, including engaging in hobbies, participating in virtual activities, and maintaining routines.

"I do genuinely believe getting out into nature has a really good impact on one's mental health and it certainly does on mine…Crafting, doing things, keeping busy…that's really important for your mental health having that occupations. I crochet… we paint, we draw, we make jewellery…"
(Participant_15_Community_mental_health_nurse)

Participants discussed the negative impact of constant news coverage on coronavirus and the death count, and intentionally restricted their news intake as they felt it was unhelpful, even harmful to their mental health:

"the virus messes with your head more than it does your body, if you're not hospitalised. That's just down to the media at the end of the day. There's so much media and so much emphasis on death, not so much on recovery" (Participant_7_Hospital_nurse)

Accepting uncertainty

Most participants had a degree of personal, psychological resilience linked to an acceptance, or 'letting go', of what they had no control over, such as the overall outcome of the pandemic or government restrictions.

"I'm generally much less anxious now than I was in January and February. And part of that, I think, is about thinking a lot more about death and being a lot more accepting about death, and about what you can control and what's out of your control" (Participant_14_GP)

For peer review only - http://bmjopen.bmj.com/site/about/guidelines.xhtml

Part of this philosophy seemed to be influenced by their previous experiences, and their profession:

“Because of my job, I think I’m aware that we’re not really in control of lots of things in our life… I see that all the time with patients and people I care for… we don’t have control over everything and we have to have a level of acceptance for that” (Participant_15_community_mental_health_nurse)

Increased sense of purpose and reward

Participants often expressed gratitude for being able to continue working. This brought with it benefits such as purpose, daily structure, predictability, a degree of socialising and being ‘in-the-know’ about the virus. Participants also talked about how it felt good to be able to contribute. This was highly rewarding and brought a heightened sense of purpose.

“…some people would view me as not being lucky, because I’m a frontline worker, but that’s my job and that’s what I’m trained to do, so I don’t view that as unlucky… I have been lucky in the sense that I’ve been able to keep busy, to keep working, to feel I’m contributing” (Participant_17_GP)

Guilt occurred when participants weren’t able to contribute as much as they would have liked e.g. they were told they couldn’t work because they were high-risk, or had to work from home.

“I felt incredibly guilty by the fact that I wasn’t helping out on the frontline because I was pregnant. I was just told I’m not allowed to see any patients, by occupational health, and sent home” (Participant_6_Hospital_doctor)

Personal growth

The pandemic also brought with it opportunities for personal growth among participants. This involved increase self-reflection, the opportunity to ‘slow down’ more outside of work, and a perceived improvement in non-work relationships.

Increased reflection

Participants reported that they were able to reflect more on “what matters” in their lives. Commonly this was spending quality time with friends and family and appreciating the small things in life.

“one benefit is that actually we quite like a simple life and actually you come to appreciate the very simple things, which are just being outside, going for a bike ride, having a picnic…just the health and happiness of your own family is what’s important and everything else, you can generally sort out” (Participant_17_GP)

For peer review only - http://bmjopen.bmj.com/site/about/guidelines.xhtml

"I think that's one of the positive things that have come out of this whole virus, it that it's allowing people to take a lot more time to reflect on themselves…to reflect on what are the things that really matter, what do I really value in life?" (Participant_10_social_worker)

Slowing down

Participants discussed how the pandemic had given them a chance to slow down and have more "me-time" and expressed this was something they wished to take forward beyond the pandemic. This view may seem in conflict with the increased workload experienced by certain participants, but reflected the changes outside of work, e.g. having fewer social obligations due to social distancing restrictions.

"We have really busy lives generally, and we spend a lot of time rushing around doing lots of stuff, and actually this time has been quite nice in many ways as a period to kind of slow down a bit and I think just appreciating each other" (Participant_1_Hospital_doctor)

"I think it has definitely made me realise that doing less is better for me in terms of not trying to have a finger in every single pie. So I hope I will be able to retain that side of it" (Participant_4_Hospital_doctor_palliative_care_registrar)

Improved non-work relationships

Whilst not unanimous, participants talked about how some of their relationships with others improved during the pandemic, especially with family members they lived with, the crisis having brought them closer.

"I've connected with my family a lot more… I feel really good about spending more time with my daughter…that's time that I would never have had with her, so that's really special" (Participant_5_academic_respiratory_physiotherapist)

"As a family, I think we've definitely become closer… we've managed to do some things together which we normally wouldn't do" (Participant_11_Family_support_worker)

Discussion

This qualitative interview study explored the psychosocial impact of the COVID-19 pandemic on health and social care professionals in the UK. We identified five key themes shared between professionals' accounts. The main difficulties reported were 'communication challenges' (consisting of 'virtual consulting' and 'difficult conversations') and 'work-related stressors' (consisting of 'need to protect loved ones', 'public not following rules' and 'increased workload and changing work conditions'). Three factors appeared to mitigate some of the psychological distress of the pandemic: 'support structures' (consisting of 'team unity', 'leadership' and 'social support'), 'resilience' (consisting of 'proactive coping', 'accepting uncertainty' and 'increased sense of purpose

For peer review only - http://bmjopen.bmj.com/site/about/guidelines.xhtml

and reward') and 'personal growth' (consisting of 'slowing down', 'increased reflection' and 'improved relationships').

The themes were drawn from interviews with professionals working in different areas of health and social care, and some themes were felt more strongly in certain jobs than others. For example, GPs and social workers enjoyed the efficiency of new virtual consultations, but GPs found it difficult to use with older adults and those with age-related cognitive decline. Those working with mental health clients, such as psychotherapists and social workers, experienced digital connectivity issues when communicating with vulnerable clients, leading to frustrating repetitions and difficulties building a trusting relationship. This corroborates findings from previous research on barriers to virtual consulting (29). Those working with older adults, found one of the most challenging elements of the pandemic was having difficult conversations over the telephone with loved ones of patients who were dying - often having to convey that visiting restrictions meant they would be unable to say goodbye in person. This is potentially concerning for the well-being of patients and healthcare professionals, given previous research has highlighted the importance of an appropriate physical and social setting in breaking bad news, and also the presence of family members (30,31).

There were a number of common work-related stressors among participants. One of these was frustration with members of the public not following the social distancing and hygiene regulations, and with the government's handling of the pandemic. Some also expressed frustration at the 'culture of blame' that they felt permeated the media and public discourse, which can be maladaptive and harmful for one's own mental well-being (32). Emotional and physical fatigue were also common experiences across all professions, corroborating qualitative studies of health and care workers during COVID-19 and previous pandemics globally (4,5,33). Many participants were worried about putting their loved ones at risk of catching COVID-19 as highlighted by previous research (34), however in our study it appears to have been a particular concern amongst carers and hospital doctors/nurses, perhaps due to their higher level of virus exposure.

Most participants reported an increased sense of team unity; that they were 'all in this together' fighting a common enemy. This may have led to a degree of resilience against some of the stressful elements of working in a pandemic, supporting findings from previous pandemics (5,10). Moreover, supporting Berkman's social networks theory (35) which informed part of our interview guide, strong social relationships were frequently cited by participants as key supportive mechanisms for their mental health during this period, including supportive partners at home, friends, family and colleagues. Those participants that did experience loneliness at work during the pandemic were lone workers, working from home, or described unsupportive management. In line with our findings, a recent systematic review of quantitative studies examining the impact of COVID-19 on healthcare workers found social support to be a vital resource underlying their ability to cope (21).

For peer review only - http://bmjopen.bmj.com/site/about/guidelines.xhtml

Participants also described using their own internal resilience as a way to buffer many of the key stressors involved in working through the pandemic, and even thrive at times. They adopted proactive coping mechanisms, as seen in healthcare workers during SARS (36). These often involved partaking in activities that now have substantial evidence for their role in improving mental health including: exercise (37), arts and crafts (38), spending time in nature (39,40), virtual activities with friends, and maintaining a healthy routine (41,42). Consistent with research from previous pandemics and recent quantitative data during COVID-19, participants also restricted their news intake, particularly as they felt the constant reporting of COVID-19 and the prevalent discourse of blame negatively impacted their mental health (6,23).

Most participants said they were successfully able to 'let go' of aspects of the pandemic that they felt were out of their control, such as the overall course of the virus and government restrictions. This demonstrates a psychological theory known as 'radical acceptance' (43), and may have been responsible in part for the resilience reported by many participants, also having been identified as a successful coping strategy for healthcare workers in previous pandemics (36). Some academics have critiqued 'resilience' as a concept for its focus on individual- rather than structural-level factors (44), however, participants in the study highlighted the link between these factors, particularly the importance of social networks and social support structures at work. Most participants also expressed gratitude for being able to continue working and described a sense of increased purpose and reward for being able to contribute during the pandemic.

Common 'personal growth' themes were frequently described in participant accounts. Most participants reflected more on 'what matters' in life during this period, which included relationships with friends and family, their health and the health of loved ones, and 'appreciating the small things' in life. These findings mirror international qualitative studies looking at the psychological impact of COVID-19 and previous pandemics on health and care workers which found they experienced 'growth under pressure' (5,33) and increased gratitude and self-reflection (33). 'Growth under pressure' may be a closely linked (but slightly diluted) concept to 'post-traumatic growth' seen in individuals experiencing personal growth in the aftermath of highly challenging life crises (45). Lastly, in relation to one of the key theories which informed our interview guide, participants demonstrated a high "sense of coherence" which may have enhanced their ability to cope during stressful experiences (19). Participants spoke about 'manageability' whereby they were highly proactive in their coping mechanisms, 'comprehensibility' in their enhanced understanding of the virus and need for social distancing restrictions, and 'meaningfulness' in how they experienced a heightened sense of purpose through their contribution during the pandemic.

Strengths & Limitations

- This is the first known study in the UK to interview both health and social care professionals working in a range of settings on their experiences working through COVID-19, which we felt important as they all continued to provide vital frontline care during the pandemic. This study used a strong

For peer review only - http://bmjopen.bmj.com/site/about/guidelines.xhtml

theoretical approach to inform the topic guide, and one-to-one interviews allowed in-depth analysis of the psychosocial experiences of health and social care professionals, complementing the wider breadth of quantitative evidence. There were also some limitations. First, we interviewed a wide range of professions, which provided breadth of experience but might limit the specificity of findings. However, due to similarities in the roles of health and care professionals we felt it important to include a range of voices. Second, given the fluctuating nature of the pandemic, attitudes of health and social care professionals may change over time. This can be difficult to capture during a single interview, however we did ask questions on how their experience had progressed longitudinally. Third, our sample may have been biased towards people who had more free time to participate and so were coping better than others. However, our sample still described a number of stressful experiences during the pandemic, and it is equally possible that workers who were frustrated or stressed wished to express their views. Lastly, interviews were conducted over the phone or video which may have limited the degree to which participants felt able to express themselves, however it may also have been that some participants felt more comfortable communicating this way. Moreover, it was also necessary during the times of the pandemic and also allowed greater uptake, convenience and good regional spread.

Implications

This study has important implications for health and social care workers, managers, commissioners of services and policy makers during the ongoing pandemic and beyond. First, it highlights the key stressors experienced by health and social care professionals during the COVID-19 pandemic. Many of these echo findings from previous epidemics, but whilst this is reassuring in terms of data credibility, it highlights a concerning lack of improvement in working conditions during such emergencies over the past two decades. It is vital that the challenges identified here are addressed. Health and care professionals navigating difficult conversations via telephone or video may benefit from extra training and support at work, for example in use of the WIRE-SPIKES protocol for breaking bad news remotely (46). Further, this study provides evidence for the supportive and coping mechanisms used by workers who experienced resilience during this period. Application of coping strategies including leisure activities were common and reportedly beneficial, as were the use of mindful techniques such as expressing gratitude. This suggests that health and care professionals may benefit from regular work-based interventions providing space for such activities. Whilst such activities may feel extraneous during emergency situations, the building of resilience and positive coping outside of pandemic situations and the tackling of problems such as staff burnout will likely improve staff coping capacity in future epidemic situations. Alongside this, adequate provision for social support should be ensured, from family and friends but also via the work place e.g. through enhanced supervision or peer support. The research presented here suggests that investment into wellbeing support could play a vital role in helping health and care workers to manage emotional stress.

For peer review only - http://bmjopen.bmj.com/site/about/guidelines.xhtml

Conclusion

To the best of our knowledge, this is the first qualitative study to explore the psychosocial impact of the COVID-19 pandemic on both health and social care professionals working in different settings across the UK. Participants experienced communication challenges and changing work conditions, but also positive factors such as increased team unity, and greater reflection on what matters in their life. This study offers important evidence for continued and future disruptions caused by the COVID-19 pandemic. It also elucidates successful psychological and practical strategies deployed by health and social care professionals that could be used to support their resilience and well-being.

Funding statement

This COVID-19 Social Study was funded by the Nuffield Foundation [WEL/FR-000022583], but the views expressed here are those of the authors. The study was also supported by the MARCH Mental Health Network funded by the Cross-Disciplinary Mental Health Network Plus initiative supported by UK Research and Innovation [ES/S002588/1], and by the Wellcome Trust [221400/Z/20/Z]. DF was funded by the Wellcome Trust [205407/Z/16/Z].

Ethical approval

The study was reviewed and approved by the UCL Ethics Committee (Project ID 14895/005).

Acknowledgements

The authors would like to thank other members of the qualitative COVID-19 social study team at UCL, who helped discuss themes during the analysis stages at weekly team meetings: Dr Thomas May, Dr Anna Roberts, and Dr Joanna Dawes.

Contributors:

DF, AB and HA conceived the study and contributed to the study design. HA conducted all the interviews apart from 1, which AB conducted. HA coded all the transcriptions. AM coded 4 transcripts for cross-checking purposes. HA wrote up the manuscript. All authors (HA, DF, AM, AB) critically reviewed the manuscript and approved the final submission.

Competing interests:

None declared.

For peer review only

Data sharing statement:
Raw data is available via participant quotations and the topic interview guide is provided in Supplementary Material.

References

1. Adhikari SP, Meng S, Wu Y-J, Mao Y-P, Ye R-X, Wang Q-Z, et al. Epidemiology, causes, clinical manifestation and diagnosis, prevention and control of coronavirus disease (COVID-19) during the early outbreak period: a scoping review. Infect Dis Poverty. 2020 Mar 17;9(1):29.
2. Iacobucci G. Covid-19: UK lockdown is “crucial” to saving lives, say doctors and scientists. BMJ [Internet]. 2020 Mar 24 [cited 2020 Sep 3];368. Available from: https://www.bmj.com/content/368/bmj.m1204
3. Shaw SCK. Hopelessness, helplessness and resilience: The importance of safeguarding our trainees’ mental wellbeing during the COVID-19 pandemic. Nurse Educ Pract. 2020 Mar;44:102780.
4. McAlonan GM, Lee AM, Cheung V, Cheung C, Tsang KWT, Sham PC, et al. Immediate and sustained psychological impact of an emerging infectious disease outbreak on health care workers. Can J Psychiatry. 2007 Apr;52(4):241–7.
5. Kim Y. Nurses’ experiences of care for patients with Middle East respiratory syndrome-coronavirus in South Korea. American Journal of Infection Control. 2018 Jul 1;46(7):781–7.
6. Lee S-H, Juang Y-Y, Su Y-J, Lee H-L, Lin Yi-Hui, Chao C-C. Facing SARS: psychological impacts on SARS team nurses and psychiatric services in a Taiwan general hospital. General Hospital Psychiatry. 2005 Sep 1;27(5):352–8.
7. Kang L, Li Y, Hu S, Chen M, Yang C, Yang BX, et al. The mental health of medical workers in Wuhan, China dealing with the 2019 novel coronavirus. The Lancet Psychiatry. 2020 Mar 1;7(3):e14.
8. Lai J, Ma S, Wang Y, Cai Z, Hu J, Wei N, et al. Factors Associated With Mental Health Outcomes Among Health Care Workers Exposed to Coronavirus Disease 2019. JAMA Netw Open. 2020 Mar 23;3(3):e203976.
9. Du J, Dong L, Wang T, Yuan C, Fu R, Zhang L, et al. Psychological symptoms among frontline healthcare workers during COVID-19 outbreak in Wuhan. Gen Hosp Psychiatry [Internet]. 2020 Apr 3 [cited 2020 Oct 26]; Available from: https://www.ncbi.nlm.nih.gov/pmc/articles/PMC7194721/
10. Sun N, Wei L, Shi S, Jiao D, Song R, Ma L, et al. A qualitative study on the psychological experience of caregivers of COVID-19 patients. American Journal of Infection Control. 2020 Jun 1;48(6):592–8.
11. Xiao H, Zhang Y, Kong D, Li S, Yang N. The Effects of Social Support on Sleep Quality of Medical Staff Treating Patients with Coronavirus Disease 2019 (COVID-19) in January and February 2020 in China. Med Sci Monit. 2020 Mar 5;26:e923549-1-e923549-8.
12. Lu W, Wang H, Lin Y, Li L. Psychological status of medical workforce during the COVID-19 pandemic: A cross-sectional study. Psychiatry Res. 2020 Jun;288:112936.
13. Huang Y, Zhao N. Generalized anxiety disorder, depressive symptoms and sleep quality during COVID-19 outbreak in China: a web-based cross-sectional survey. Psychiatry Res. 2020 Jun;288:112954.

For peer review only - http://bmjopen.bmj.com/site/about/guidelines.xhtml

14. Quick TL. Linking productivity and health. Healthy Work: Stress, Productivity, and the Reconstruction of Working Life Robert Karasek and Tores Theorell New York: Basic Books, Inc. 1990 $29.95 Canada $39.95 381 pages. National Productivity Review. 1990;9(4):475–8.

15. Imo UO. Burnout and psychiatric morbidity among doctors in the UK: A systematic literature review of prevalence and associated factors. BJPsych Bulletin. 2017 Aug;41(4):197–204.

16. Coyle D, Edwards D, Hannigan B, Fothergill A, Burnard P. A systematic review of stress among mental health social workers. International Social Work. 2005 Mar 1;48(2):201–11.

17. Vindrola-Padros C, Andrews L, Dowrick A, Djellouli N, Fillmore H, Gonzalez EB, et al. Perceptions and experiences of healthcare workers during the COVID-19 pandemic in the UK. BMJ Open. 2020 Nov 1;10(11):e040503.

18. Williamson V, Murphy D, Greenberg N. COVID-19 and experiences of moral injury in front-line key workers. Occup Med (Lond). 2020 Jul 17;70(5):317–9.

19. Eriksson M. The Sense of Coherence in the Salutogenic Model of Health. In: Mittelmark MB, Sagy S, Eriksson M, Bauer GF, Pelikan JM, Lindström B, et al., editors. The Handbook of Salutogenesis [Internet]. Cham (CH): Springer; 2017 [cited 2019 Nov 27]. Available from: http://www.ncbi.nlm.nih.gov/books/NBK435812/

20. Eriksson M, Lindstrom B. Antonovsky's sense of coherence scale and its relation with quality of life: a systematic review. Journal of Epidemiology & Community Health. 2007 Nov 1;61(11):938–44.

21. Labrague LJ. Psychological resilience, coping behaviours, and social support among healthcare workers during the COVID-19 pandemic: a systematic review of quantitative studies. medRxiv. 2020 Nov 6;2020.11.05.20226415.

22. Nyashanu M, Pfende F, Ekpenyong M. Exploring the challenges faced by frontline workers in health and social care amid the COVID-19 pandemic: experiences of frontline workers in the English Midlands region, UK. Journal of Interprofessional Care. 2020 Sep 2;34(5):655–61.

23. Bu F, Steptoe A, Mak HW, Fancourt D. Time-use and mental health during the COVID-19 pandemic: a panel analysis of 55,204 adults followed across 11 weeks of lockdown in the UK. medRxiv. 2020 Aug 21;2020.08.18.20177345.

24. Tong A, Sainsbury P, Craig J. Consolidated criteria for reporting qualitative research (COREQ): a 32-item checklist for interviews and focus groups. Int J Qual Health Care. 2007 Dec 1;19(6):349–57.

25. Berkman LF. The Assessment of Social Networks and Social Support in the Elderly. Journal of the American Geriatrics Society. 1983;31(12):743–9.

26. Braun V, Clarke V. Reflecting on reflexive thematic analysis. Qualitative Research in Sport, Exercise and Health. 2019 Aug 8;11(4):589–97.

27. Braun V, Clarke V. Using thematic analysis in psychology. Qualitative Research in Psychology. 2006 Jan;3(2):77–101.

28. QSR International Pty Ltd. NVIVO [Internet]. [cited 2020 Nov 4]. Available from: https://www.qsrinternational.com/nvivo-qualitative-data-analysis-software/home

29. Donaghy E, Atherton H, Hammersley V, McNeilly H, Bikker A, Robbins L, et al. Acceptability, benefits, and challenges of video consulting: a qualitative study in primary care. Br J Gen Pract. 2019 Sep 1;69(686):e586–94.

For peer review only

For peer review only - http://bmjopen.bmj.com/site/about/guidelines.xhtml

30. Monden KR, Gentry L, Cox TR. Delivering Bad News to Patients. Baylor University Medical Center Proceedings. 2016 Jan 1;29(1):101–2.

31. Ptacek JT, Fries EA, Eberhardt TL, Ptacek JJ. Breaking bad news to patients: physicians' perceptions of the process. Support Care Cancer. 1999 Apr 1;7(3):113–20.

32. Tennen H, Affleck G. Blaming others for threatening events. Psychological Bulletin. 1990;108(2):209–32.

33. Sun N, Wei L, Shi S, Jiao D, Song R, Ma L, et al. A qualitative study on the psychological experience of caregivers of COVID-19 patients. American Journal of Infection Control. 2020 Jun 1;48(6):592–8.

34. Balasubramanian A, Paleri V, Bennett R, Paleri V. Impact of COVID-19 on the mental health of surgeons and coping strategies. Head & Neck. 2020;42(7):1638–44.

35. Berkman LF. Social Networks and Health. 2010;27.

36. Wong TW, Yau JKY, Chan CLW, Kwong RSY, Ho SMY, Lau CC, et al. The psychological impact of severe acute respiratory syndrome outbreak on healthcare workers in emergency departments and how they cope. European Journal of Emergency Medicine. 2005 Feb;12(1):13–18.

37. Stathopoulou G, Powers MB, Berry AC, Smits JAJ, Otto MW. Exercise Interventions for Mental Health: A Quantitative and Qualitative Review. Clinical Psychology: Science and Practice. 2006;13(2):179–93.

38. Fancourt D, Finn S. What is the evidence on the role of the arts in improving health and well-being? A scoping review [Internet]. WHO Regional Office for Europe, Copenhagen; 2019. (WHO Health Evidence Network Synthesis Reports). Available from: http://europepmc.org/books/NBK553773

39. Clatworthy J, Hinds J, Camic PM. Gardening as a mental health intervention: a review. Mental Health Review Journal [Internet]. 2013 Nov 29 [cited 2020 Oct 30]; Available from: https://www.emerald.com/insight/content/doi/10.1108/MHRJ-02-2013-0007/full/html

40. Kotera Y, Richardson M, Sheffield D. Effects of Shinrin-Yoku (Forest Bathing) and Nature Therapy on Mental Health: a Systematic Review and Meta-analysis. Int J Ment Health Addiction [Internet]. 2020 Jul 28 [cited 2020 Oct 30]; Available from: https://doi.org/10.1007/s11469-020-00363-4

41. Lyall LM, Wyse CA, Graham N, Ferguson A, Lyall DM, Cullen B, et al. Association of disrupted circadian rhythmicity with mood disorders, subjective wellbeing, and cognitive function: a cross-sectional study of 91 105 participants from the UK Biobank. The Lancet Psychiatry. 2018 Jun 1;5(6):507–14.

42. Lanza HI, Drabick DAG. Family Routine Moderates the Relation Between Child Impulsivity and Oppositional Defiant Disorder Symptoms. J Abnorm Child Psychol. 2011 Jan 1;39(1):83–94.

43. Robins CJ, Schmidt III H, Linehan MM. Dialectical Behavior Therapy: Synthesizing Radical Acceptance with Skillful Means. In: Mindfulness and acceptance: Expanding the cognitive-behavioral tradition. New York, NY, US: Guilford Press; 2004. p. 30–44.

44. Fletcher D, Sarkar M. Psychological resilience: A review and critique of definitions, concepts, and theory. European Psychologist. 2013;18(1):12–23.

45. Tedeschi RG, Calhoun LG. Posttraumatic Growth: Conceptual Foundations and Empirical Evidence. Psychological Inquiry. 2004 Jan 1;15(1):1–18.

46. Holstead RG, Robinson AG. Discussing Serious News Remotely: Navigating Difficult Conversations During a Pandemic. JCO Oncology Practice. 2020 May 18;16(7):363–8.

For peer review only

For peer review only

For peer review only - http://bmjopen.bmj.com/site/about/guidelines.xhtml

- How would you describe your social life now that social distancing measures have been brought in because of Covid-19?
- In what ways has your work life been impacted by the Covid-19 pandemic?
- How do you feel about the changes that have been brought about by Covid-19? Have they had any impact on your mental health or wellbeing?
- Have there been any positive experiences for you resulting from the Covid-19 pandemic?

Figure 1: Examples of Questions in the Topic Guide

For peer review only

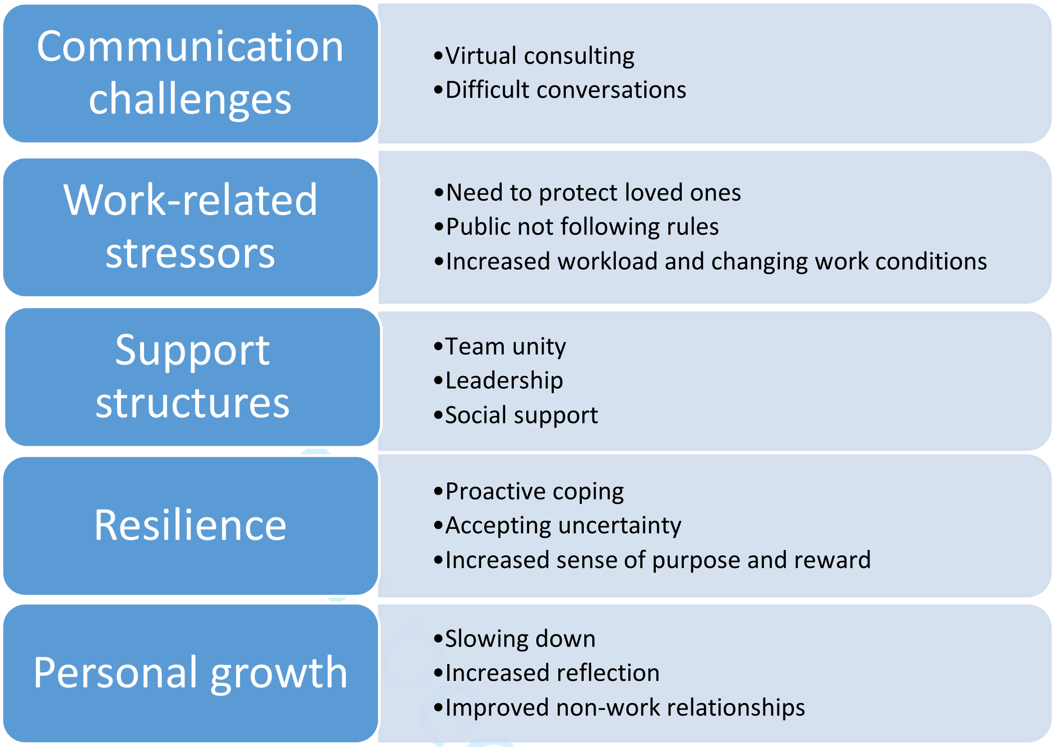

Figure 2: Themes and sub-themes

Draft topic guide: Health and Care Workers

Ask to describe 'normal life' – before the crisis, and now

- Employed? Type of job, hours etc,
- Full time parent or carer?
- Who you normally live with, does this change, separated/ extended family?
- Whether you would usually have done any type(s) of regular exercise (whatever they perceive as exercise including walking/gardening)

SOCIAL LIFE

What was your social life before the Covid-19 pandemic? Has this changed? If so, what has been the impact of Covid-19 on your social life?

- How would you describe your social network before Covid – for example size, types of people, types of relationships, do they live with you, nearby or further away, how often do you see each other, how well do you know each other? How do you interact, face to face, online or social media? Describe some of your common socialising activities. **Has this changed? What has the impact of Covid been on your social network?**
- Can you tell us about any ways your social networks/ friendship groups influence you, such as peer pressure, or encouraging you to get involved in things? Do you compare your life to theirs?
- Could you describe any community participation or volunteering participation before Covid? **Has this changed? If so, what has been the impact of Covid-19 on community participation/volunteering participation?**
- Could you describe the social support you have before Covid? (such as emotional support, advice and information, someone to help you with money or milk/bread/essentials) **Has this changed? If so, what has been the impact of Covid-19 on your social support?**
- **Social engagement (social roles, bonding, attachment) (pre- and post- Covid)**

WORK LIFE

How would you describe your work life before the Covid-19 pandemic?

Prompts include:

- Describe a typical day?
- Describe your work environment prior to the crisis
- How much autonomy did you have in your role?
- Did you find your job rewarding?
- Did you feel able to do your job to a high standard?
- Did you enjoy your job?
- Describe your sense, if any, of team unity or disunity prior to this crisis?
- How able were you to follow organisational rules and how did you feel about this?
- Normally did you feel safe at work? In what way?

How would you describe your work life since the Covid-19 pandemic? Please tell us about this

For peer review only - http://bmjopen.bmj.com/site/about/guidelines.xhtml

- Describe a typical day now – how have common work practices changed? Have you adapted your work in response to Covid-19 (e.g. delivery, operating hours, change of products/production methods)
- Describe your overall work environment now
- How much autonomy do you feel you have at the moment and how has this changed?
- Are you finding work rewarding at the moment?
- Do you feel able to do your job to a high standard – has this changed since the crisis?
- Enjoyment – do you currently enjoy your job?
- Describe your sense, if any, of team unity or disunity during this crisis?
- How able are you to follow organisational rules and how do you feel about this?
- Do you feel safe? If this has changed, how?

MENTAL HEALTH

How do you feel about the changes that have been brought about by Covid-19? Have they had any impact on your mental health or wellbeing? Please tell us about these

- What are the things most bothering you at the moment (work or outside of work)?
- What have been the major triggers/causes of any mental health or wellbeing issues?
- How have government guidelines or organisational guidelines impacted your mental health or wellbeing?
- Have you experienced any impact on positive emotions? (prompts: how deeply you can engage with what you are doing, sense of meaning/ purpose, relationships with others, how well you are managing and feelings of control over your situation?)
- Has there been any impact on your sense of identity?
- Have you experienced any negative psychological feelings? (prompts: such as shame, guilt, lack of pleasure, anxiety, worry)
- Please tell us about any physical symptoms due to being stressed or anxious? (prompts: fatigue, sleep problems, pain, illness symptoms, palpitations)

Have you been doing/ planning anything to help with this?

- How has your support been, from friends/family? From work colleagues/your organisation?
- Connecting with family or friends online
- Online groups?
- Hobbies/ Reading
- Exercise at home <ask about what they have been doing and if there are specific resources they have found useful to exercise>
- Volunteering
- Other engagement

Why are you doing/ not doing these things?

- Helpful/ not helpful – please tell us why
- Enjoyable
- Good for mental health/ wellbeing
- Can't get online, not connected, not comfortable, affordability, confidence in using/ skills
- Skills in using the internet/ communication software
- Living arrangements/ Work/ caring demands
- Peer support/ pressure
- Difficulties/ restriction in physical environment

PROSPECTION

For peer review only - http://bmjopen.bmj.com/site/about/guidelines.xhtml

Has the pandemic meant that you have any worries for the future?

- Worries about work/the future of your work?
- Worries for yourself? Anything not directly connected to work?

How are these different from the worries you had before?

- Sense of control/ powerlessness
- Severity of worries / perspective

Will this change the way you live your life in future?

- The way you connect with others
- How you look after yourself
- How you support others
- How you exercise?

Do you think there will be any changes to the way you work in the future? Why/why not?

Has this changed any of your priorities for the future?

For peer review only

For peer review only - http://bmjopen.bmj.com/site/about/guidelines.xhtml

COREQ (COnsolidated criteria for REporting Qualitative research) Checklist

A checklist of items that should be included in reports of qualitative research. You must report the page number in your manuscript where you consider each of the items listed in this checklist. If you have not included this information, either revise your manuscript accordingly before submitting or note N/A.

Topic	Item No.	Guide Questions/Description	Reported on Page No.
**Domain 1: Research team and reflexivity**			
Personal characteristics			
Interviewer/facilitator	1	Which author/s conducted the interview or focus group?	
Credentials	2	What were the researcher's credentials? E.g. PhD, MD	
Occupation	3	What was their occupation at the time of the study?	
Gender	4	Was the researcher male or female?	
Experience and training	5	What experience or training did the researcher have?	
Relationship with participants			
Relationship established	6	Was a relationship established prior to study commencement?	
Participant knowledge of the interviewer	7	What did the participants know about the researcher? e.g. personal goals, reasons for doing the research	
Interviewer characteristics	8	What characteristics were reported about the inter viewer/facilitator? e.g. Bias, assumptions, reasons and interests in the research topic	
**Domain 2: Study design**			
Theoretical framework			
Methodological orientation and Theory	9	What methodological orientation was stated to underpin the study? e.g. grounded theory, discourse analysis, ethnography, phenomenology, content analysis	
Participant selection			
Sampling	10	How were participants selected? e.g. purposive, convenience, consecutive, snowball	
Method of approach	11	How were participants approached? e.g. face-to-face, telephone, mail, email	
Sample size	12	How many participants were in the study?	
Non-participation	13	How many people refused to participate or dropped out? Reasons?	
Setting			
Setting of data collection	14	Where was the data collected? e.g. home, clinic, workplace	
Presence of non-participants	15	Was anyone else present besides the participants and researchers?	
Description of sample	16	What are the important characteristics of the sample? e.g. demographic data, date	
Data collection			
Interview guide	17	Were questions, prompts, guides provided by the authors? Was it pilot tested?	
Repeat interviews	18	Were repeat inter views carried out? If yes, how many?	
Audio/visual recording	19	Did the research use audio or visual recording to collect the data?	
Field notes	20	Were field notes made during and/or after the inter view or focus group?	
Duration	21	What was the duration of the inter views or focus group?	
Data saturation	22	Was data saturation discussed?	
Transcripts returned	23	Were transcripts returned to participants for comment and/or	

For peer review only

Topic	Item No.	Guide Questions/Description	Reported on Page No.
		correction?	
**Domain 3: analysis and findings**			
Data analysis			
Number of data coders	24	How many data coders coded the data?	
Description of the coding tree	25	Did authors provide a description of the coding tree?	
Derivation of themes	26	Were themes identified in advance or derived from the data?	
Software	27	What software, if applicable, was used to manage the data?	
Participant checking	28	Did participants provide feedback on the findings?	
Reporting			
Quotations presented	29	Were participant quotations presented to illustrate the themes/findings? Was each quotation identified? e.g. participant number	
Data and findings consistent	30	Was there consistency between the data presented and the findings?	
Clarity of major themes	31	Were major themes clearly presented in the findings?	
Clarity of minor themes	32	Is there a description of diverse cases or discussion of minor themes?	

Developed from: Tong A, Sainsbury P, Craig J. Consolidated criteria for reporting qualitative research (COREQ): a 32-item checklist for interviews and focus groups. *International Journal for Quality in Health Care*. 2007. Volume 19, Number 6: pp. 349 – 357

Once you have completed this checklist, please save a copy and upload it as part of your submission. DO NOT include this checklist as part of the main manuscript document. It must be uploaded as a separate file.

For peer review only
